# Pharmacogenetics of metamizole-induced agranulocytosis: a systematic review and drug regulation implications

**DOI:** 10.3389/fphar.2025.1624044

**Published:** 2025-09-24

**Authors:** Giovana Fernanda Santos Fidelis, Carolina Dagli-Hernandez, Romina P. Martinelli, Jefman Efendi Marzuki, Baharuddin Baharuddin, Qun-Ying Yue, Brian Edwards, Eder de Carvalho Pincinato, Patricia Moriel

**Affiliations:** ^1^ School of Medical Sciences, Universidade Estadual de Campinas, Campinas, Brazil; ^2^ School of Pharmaceutical Sciences, Universidade de São Paulo, Sao Paulo, Brazil; ^3^ Faculty of Pharmaceutical Sciences, Universidade Estadual de Campinas, Campinas, Brazil; ^4^ Delta PV, Spanish Team, Madrid, Spain; ^5^ Faculty of Medicine, Universitas Surabaya, Surabaya, Indonesia; ^6^ Ubaya Hospital, Surabaya, Indonesia; ^7^ Uppsala Monitoring Centre, Uppsala, Sweden; ^8^ International Society of Pharmacovigilance, Geneva, Switzerland

**Keywords:** metamizole, dipyrone, agranulocytosis, pharmacogenetics, regulation

## Abstract

**Introduction:**

Metamizole (dipyrone) is a widely used analgesic and antipyretic, but its use has been restricted in some countries due to the risk of metamizole-induced agranulocytosis (MIA). This systematic review investigated genetic variants associated with MIA, allele frequencies across ancestry groups, and the drug's legal status.

**Methods:**

A literature search was conducted across nine databases for studies published up to 08 April 2025. Study selection and data extraction were performed by two independent reviewers. The Withdrawn 2.0 platform was used to assess the global legal status of metamizole, while allele frequencies were obtained from the gnomAD browser (version v4.1.0) and the Allele Frequency Net Database (AFND). Bibliometric analysis was conducted using the VOS viewer software.

**Results:**

In total, four studies were included in the review. Data related to HLA, NAT2, CYP2C9, CYP2C19, and variants located on chromosome nine were reported; however, statistically significant associations were observed only for variants in chromosome nine and HLA-C*04:01. When comparing countries from different continents with varying metamizole status, the analysis of allele frequencies did not reveal sufficient differences in allele frequencies between countries, which does not justify distinct regulatory frameworks.

**Conclusion:**

This study highlights the scarcity of data in the literature reporting the association between genetic variants and MIA. Furthermore, there is insufficient evidence to justify the prohibition of metamizole in certain countries based on genetic variants alone. Additional studies are essential to evaluate the prevalence of MIA, better characterize populations, and explore potential genetic associations, particularly concerning the HLA-C*04:01 allele.

**Clinical Trial Registration:**

https://www.crd.york.ac.uk/PROSPERO/view/CRD42024572038, identifier CRD42024572038.

## Introduction

Adverse drug reactions (ADR) represent a relevant problem in public health. Even though some of them could be inoffensive, others can be life-threatening. Among a wide variety of ADRs, blood disorders, despite being rare, are associated with significant morbidity and mortality. One of the most prevalent hematologic disorders triggered by drugs is agranulocytosis (DiPiro et al., 2023), and in this case is called drug-induced agranulocytosis.

Agranulocytosis is an extreme form of neutropenia, defined as a circulating blood neutrophil number lower than 500/µL (Gibson and Berliner, 2014). This condition could begin as asymptomatic or manifest with general symptoms like fever or chills. Decreased neutrophils lead to infections, which can often be serious and result in sepsis.

The pathophysiological mechanisms underlying drug-induced agranulocytosis are not yet fully elucidated, and the current understanding remains limited, as much of the available evidence stems from scarce or outdated studies, many of which rely on *in vitro* models or cell lines rather than primary patient samples. Nonetheless, two general pathways have been proposed. One of these mechanisms involves direct cytotoxicity, which is often associated with cancer or immunosuppressive therapy and typically occurs in a dose-dependent manner. An example of this kind of drug is methotrexate, which is associated with bone marrow suppression (Hamed et al., 2022). On the other hand, non-cytotoxic drugs may trigger an idiosyncratic mechanism. These reactions, also known as type B, are mediated by hypersensitivity mechanisms and typically show no consistent correlation with dosage (Johnston and Uetrecht, 2015).

Metamizole, also known as dipyrone, is a potent analgesic and antipyretic drug broadly used in medical practice that has agranulocytosis among its side effects. The mechanism of metamizole-induced agranulocytosis (MIA) appears to involve the formation of drug-dependent anti-neutrophil antibodies that necessitate the covalent attachment of neutrophils to metamizole and its metabolites (Lutz, 2019). However, other studies indicate that the primary metamizole metabolite, N-methyl-4-aminoantipyrine, may exert a direct toxic effect on granulocyte precursors when combined with hemin (Rudin et al., 2019a; Rudin et al., 2019b).

In the last few years, the study of genetic polymorphisms has gained importance in understanding individual susceptibility to drug-induced agranulocytosis, including that caused by metamizole. Variations in genes related to drug metabolism, immune response, and neutrophil function have been identified as potential contributors to the idiosyncratic nature of this condition. These genetic insights are crucial for identifying at-risk populations and potentially guiding safer use of the drug in clinical practice.

Estimates of the risk of MIA are relatively low. The incidence was estimated for different populations as 0.96 cases per million per year in Germany (Huber et al., 2015), 1.36 per million in the Netherlands (van der Klauw et al., 1999; Basak et al., 2010), 0.7 cases per million adults per year in Poland, and 0.46 to 1.63 per million person-days in Switzerland (Blaser et al., 2015). A recent study carried out in Spain reported 1–10 cases per million users (Maciá-Martínez et al., 2024). The incidence seems even lower in non-European countries. In the LATIN study, which recruited patients in Mexico, Argentina, and Brazil, the estimated incidence for agranulocytosis was 0.38 per 1 million inhabitant-years, and no significant association was found between agranulocytosis and metamizole exposure in the previous 10 days (Hamerschlak et al., 2008).

Although the risk of agranulocytosis is not exceptionally high, the seriousness of the clinical presentation raises doubts about the benefit-risk balance. Nowadays, metamizole is widely used around the world. However, some countries have withdrawn their licenses due to safety concerns. For instance, Canada and the U.S. banned metamizole in 1963 and 1977, respectively. Subsequently, the same happened in other countries such as the U.K., France, Sweden, Norway, and Australia. However, in other countries from Europe, Asia and South America, it is still available (Cascorbi, 2021).

Considering the distinct regulatory frameworks across countries, the investigation of at-risk populations and the intrinsic characteristics of different ancestry groups emerges as a crucial factor in understanding such disparities. Therefore, the purpose of this systematic review was to map out and summarize scientific evidence regarding associations between genetic variants and the development of MIA.

## Materials and methods

This review followed the Preferred Reporting Items for Systematic Reviews and Meta-Analyses statement (PRISMA) 2020 checklist and reporting guideline (Page et al., 2021) (Supplementary Table S1). This study was registered with the International Prospective Register of Systematic Reviews (PROSPERO) (registration number CRD42024572038).

### Search strategy

A comprehensive literature search was conducted to identify relevant studies published before 08 April 2025, to answer the following question: Are there any studies in the literature that have investigated the association between genetic variants and the development of MIA? The search strategy involved consulting seven databases: PubMed, BVS/BIREME, EBSCOHOST, Scopus, Web of Science, Embase, and PROQUEST. In addition to the databases, Google Academic was consulted for further records. We limited the search to the first 15 pages as a strategy to prioritize higher-quality evidence by excluding grey literature, which peaks around pages 20–30 according to previous studies (Haddaway et al., 2015). The full search strategy for all databases is presented in Supplementary Table S2. The search was restricted to human-subject studies published in English or Spanish, due to a lack of resources to review and code studies published in other languages. Duplicated studies were excluded from the analysis.

### Study selection

The PICOS framework (population, intervention, comparator, outcomes, and study design) was applied to guide study selection: P (population): individuals exposed to metamizole; I (intervention): presence of genetic variants potentially associated with MIA; C (comparison): individuals not carrying the respective variant or general population data (when available); O (outcome): development of MIA; S (study design): observational (namely, cohort, case-control, and cross-sectional). Accordingly, studies that analyzed the association between genetic variants and MIA in patients of all ages were included. Non-human or *in vitro* studies, literature reviews, expert opinion papers, letters to the editor, commentaries, abstracts, preprints and ecological studies were excluded.

All retrieved papers were uploaded to Rayyan^®^ software and duplicates were excluded. Two reviewers (C.D.H. and G.F.S.F.) independently screened the titles and abstracts to identify potentially relevant studies according to the inclusion and exclusion criteria. Furthermore, they independently reviewed the full-text articles according to the inclusion criteria. Any discrepancies were resolved by a third reviewer (P.M.).

### Data extraction

Data extraction was performed independently by two reviewers (C.D.H. and G.F.S.F.) and any discrepancies were resolved by a third reviewer (P.M.). The data were extracted from the studies included using Microsoft Excel^®^ 2016, and the following information was extracted: the name of the first author and year of publication, country (ies) where the study was conducted, study design (retrospective or prospective), sample size, male percentage, mean age, study population, treatment duration, funding sources/sponsors, genotyping method, genes, SNPs studied and its allelic frequency.

### Data collections

To ascertain the legal status of metamizole on a global scale, searches were conducted on the Withdrawn 2.0 platform (https://bioinformatics.charite.de/withdrawn_3/index.php) (last update in October 2023) and in research articles (van der Klauw et al., 1999; Hamerschlak et al., 2008; Guimarães et al., 2021; Eleutério et al., 2024). This platform offers a repository of drug-related data, including information on drug mechanisms of action and pharmacovigilance data from the FDA Adverse Event Reporting System (FAERS). Moreover, the platform provides an overview of withdrawn drugs, with emphasis on elucidating their adverse effects and the underlying reasons for withdrawal (Gallo et al., 2024).

The allele frequencies of the genes in world populations were obtained using the software’s described below. The allele frequencies of SVEP1 rs55898176 and rs4427239 were obtained via the gnomAD browser, version v4.1.0 (https://gnomad.broadinstitute.org/) (accessed on 1 April 2025), and allele frequencies of HLA were obtained via the Allele Frequency Net Database (AFND) (https://www.allelefrequencies.net/default.asp).

### Quality assessment

The quality assessment was conducted according to the Newcastle-Ottawa Quality Assessment Scale (NOS), which was carried out by two reviewers (G.F.S.F. and C.D.H.). Any disagreements were resolved by discussion with the third reviewer (P.M.).

The NOS was designed to evaluate the methodological quality of studies, particularly cohort and case-control studies. It comprises three dimensions: selection, comparability, and outcome (in cohort studies) or exposure (in case-control studies). Each item within domain is rated with a star if the study meets predefined criteria, such as the adequacy of case definition, representativeness of the sample, or appropriate control for co-founding factors. The total score ranges from zero to nine stars, with maximum scores of four stars to the selection domain, two for comparability, and three for outcome and exposure (Stang, 2010). In this review, we adopted a general classification of the NOS, which is as follows: high quality (≥7 stars), moderate quality (4–6 stars), or low quality (<4 stars).

### Bibliometric analysis

Bibliometric analysis was employed to explore the relative relationships between studies (topics) and to identify trends and developments within clusters (Williams et al., 2019). Additionally, this analysis plays an important role in understanding the direction and strategies of a country in the context of precision medicine related to metamizole. This is a scientific method for deep analysis in health reference data (Manoj Kumar L. et al., 2023). In this study, a visualization approach was applied to the selected scientific references. Unit analysis approach focused on keyword co-occurrence. The interpretation was based on node size, relationships between nodes, and colors, which represented temporal relationships. The bibliometric analysis was conducted by (B.B.) and (J.E.) using VOS viewer software. The image resolution improved by using R software.

## Results

### Search results

**FIGURE 1:**
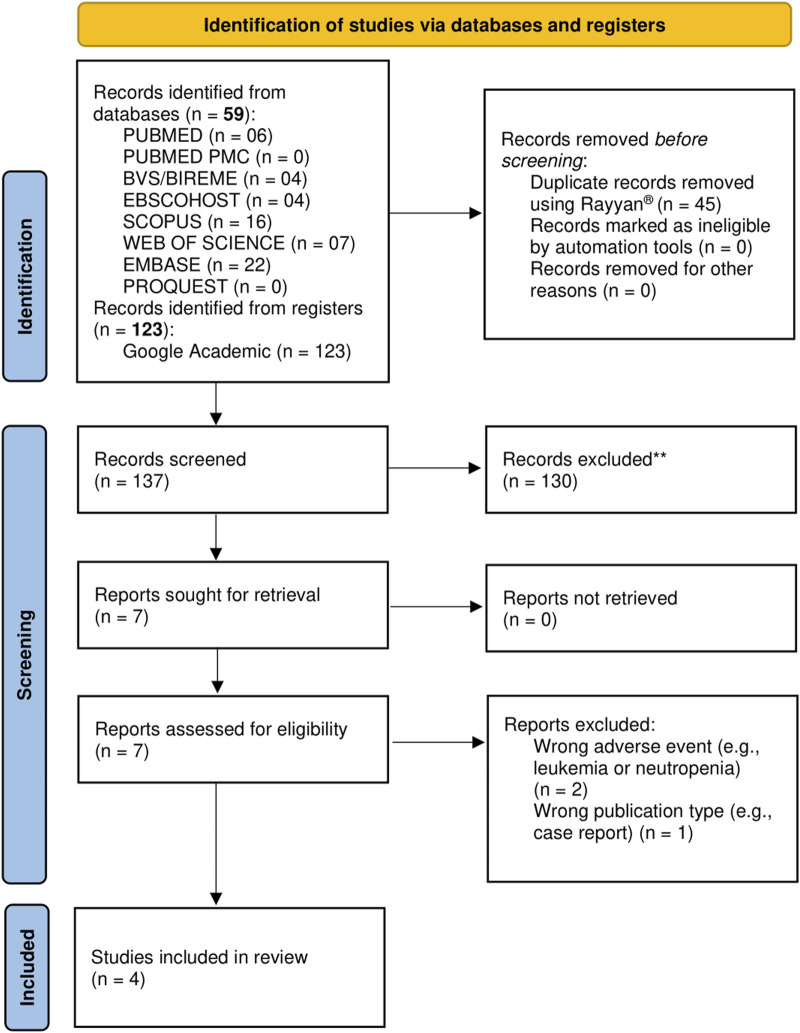
PRISMA flow diagram of study selection. The diagram details the number of records identified through databases and registers searches, the number of duplicates removed, records screened, full-text articles assessed for eligibility, reasons for full-text exclusions, and studies included in the final systematic review, in accordance with PRISMA 2020 guidelines.

The flowchart of the literature search process is shown in Figure 1. A total of 182 studies were identified from the databases and registers, of which 7 studies were selected for full-text analysis after the removal of duplicates and screening based on the title and abstract. Of these, only four studies met the inclusion criteria for this review. The excluded studies and the exclusion criteria are detailed in Supplementary Table S3.

### Characteristics of studies, participants, and genes

The characteristics of the four included studies are listed in Table 1. The studies were performed between 1996 and 2021 and conducted in Germany (Radulovic et al., 2021), Bulgaria (Vlahov et al., 1996), and in multicenter settings (Switzerland, Germany, and Spain) (Cismaru A. et al., 2020; Cismaru et al., 2020). All studies had retrospective cohorts. Only one was a cohort study, and the remaining were case-control studies.

**TABLE 1:** Characteristics of included studies.

Author/year	Country	Study design	Sample size (n)	Sample size (male, %)	Age, mean ± SD or median (range)	Population	Latency time*^a^/treatment duration^b^, days	Funding sources/sponsors
Vlahov/1996 (Vlahov et al., 1996)	Bulgaria	Retrospective (CC)	Cases (*n =* 9) (5 previously exposed to metamizole) Controls (*n =* 180)	Cases: 5 (55.6) Controls: 96 (53.3)	Cases: 45 ± 12.5 Controls: 34 ± 10.2	*Cases:* Patients who developed agranulocytosis *Controls:* healthy, unrelated subjects from the Bulgarian population	NR	NR
Cismaru/2020a, 2020b^c^ (Cismaru et al., 2020b ; 2020a)	Swiss Germany Spain	Retrospective (CC)	*Swiss* Cases (*n =* 45) Controls (tolerant and unexposed controls) (*n =* 191) *Germany* Cases (*n =* 12) Controls (*n =* 92) *Spain* Cases (*n =* 29) Controls (*n =* 145)	*Swiss* Cases: 13 (42) Controls: 17 (45) *Germany* Cases: 4 (33) Controls: 41 (44.5) *Spain* Cases: 6 (21) Controls: 87 (48)	*Swiss* Cases: 24 (19–47) Controls: 28 (16–39) *Germany* Cases: NR Controls: NR *Spain* Cases: NR Controls: NR	*Cases:* metamizole-induced agranulocytosis patients *Controls:* unexposed to metamizole *Tolerants:* patients who had received at least 500 mg of metamizole per day for at least 28 consecutive days	*Swiss* Cases: 17 (1–204) Controls: 25 (1–5,297) *Germany* Cases: 33.5 (4–9,855) Controls: NR *Spain* Cases: 11.5 (1–235) Controls: NR	Swiss National Science Foundation, Carlos III Spanish Health Institute, European Regional, Development Fund FEDER, Swedish Research Council, Swedish Heart and Lung Foundation Federal Institute for Drugs and Medical Devices, EC 5th Framework program, Serious Adverse Events Consortium (SAEC), National Institute for Health Research, Biomedical Research Center, National Health Service Foundation Trust, King’s, College London
Radulovic/2021 (Radulovic et al., 2021)	Germany	Retrospective (C)	3	0 (0)	17 (17–17)	Patients who developed severe agranulocytosis after metamizole intake	NR	NR

Abbreviations: C, cohort study; CC, case–control study; NR, not reported; *latency missing for 3 MIA, cases and 1 MIN, case.

^a^ Cases.

^b^ Controls.

^c^ The studies Cismaru A. L. et al. (2020) and Cismaru A. et al. (2020) have the same cohort.

The sample size ranged from 3 to 236 participants, and ages from 17 to 78, demonstrating the variability found in the studies. All studies included patients who developed agranulocytosis as cases, defined as absolute neutrophil count (ANC) below 0.5 × 10^9^ cells/L. Patients who had never been exposed to metamizole or who were described as “healthy and unrelated” were considered controls. The multicenter studies included tolerant patients, defined as those who received at least 500 mg of metamizole per day for at least twenty-eight consecutive days. This category of patients was considered as controls in subsequent analyses.

### Quality assessment

The methodological quality of the studies was evaluated according to the NOS, presented in Table 2. As previously stated, this review employs a general NOS classification, which is as follows: high quality (≥7 stars), moderate quality (4–6 stars), or low quality (<4 stars).

**TABLE 2:** Methodological quality of the studies included in this systematic review based on the Newcastle-Ottawa Scale.

Author, year	Study design	Selection				Comparability	Outcome/Exposure			Total score
		Item 1	Item 2	Item 3	Item 4	Item 1	Item 1	Item 2	Item 3	
Vlahov/1996 (Vlahov et al., 1996)	Retrospective (CC)	*		*	*		*	*		5
Cismaru/2020a (Cismaru et al., 2020a)	Retrospective (CC)	*		*	*		**	*	*	7
Cismaru/2020b (Cismaru et al., 2020b)	Retrospective (CC)	*		*	*		**	*	*	7
Radulovic/2021 (Radulovic et al., 2021)	Retrospective (C)		*				*	*	*	4

C, cohort study; CC, case–control study; Case-controls, Selection - Item 1, Is the case definition adequate? Item 2, Representativeness of the Cases; Item 3, Selection of Controls; Item 4, Definition of Controls. Comparability - Item 1, Comparability of Cases and Controls on the Basis of the Design or Analysis. Outcome/Exposure - Item 1, Ascertainment of Exposure; Item 2, Non-Response Rate; Item 3, Non-Response rate. Cohort studies, Selection - Item 1, representativeness of the exposed cohort; Item 2, selection of the non-exposed cohort; Item 3, ascertainment exposure; and Item 4, demonstration that the outcome was not present at the start of the study. Comparability—Item 1, comparability of cohorts. Outcome/Exposure—Item 1, assessment of outcome. Item 2, follow-up of outcomes; and Item 3, adequacy of follow-up of cohorts. The total scores ranged from four to seven stars. * One star; ** two star.

The total scores ranged from four to seven stars. The studies with the highest scores were (Cismaru A. L. et al., 2020; Cismaru et al., 2020), which received a total of seven stars, classified as high quality. The studies conducted by Radulovic et al. (2021) and Vlahov et al. (1996) were classified as moderate quality, with a total of four and five stars, respectively. In the comparability domain, which assesses cofounding factors in case-control studies, none of the included studies received stars.

### Bibliometric analysis

**FIGURE 2:**
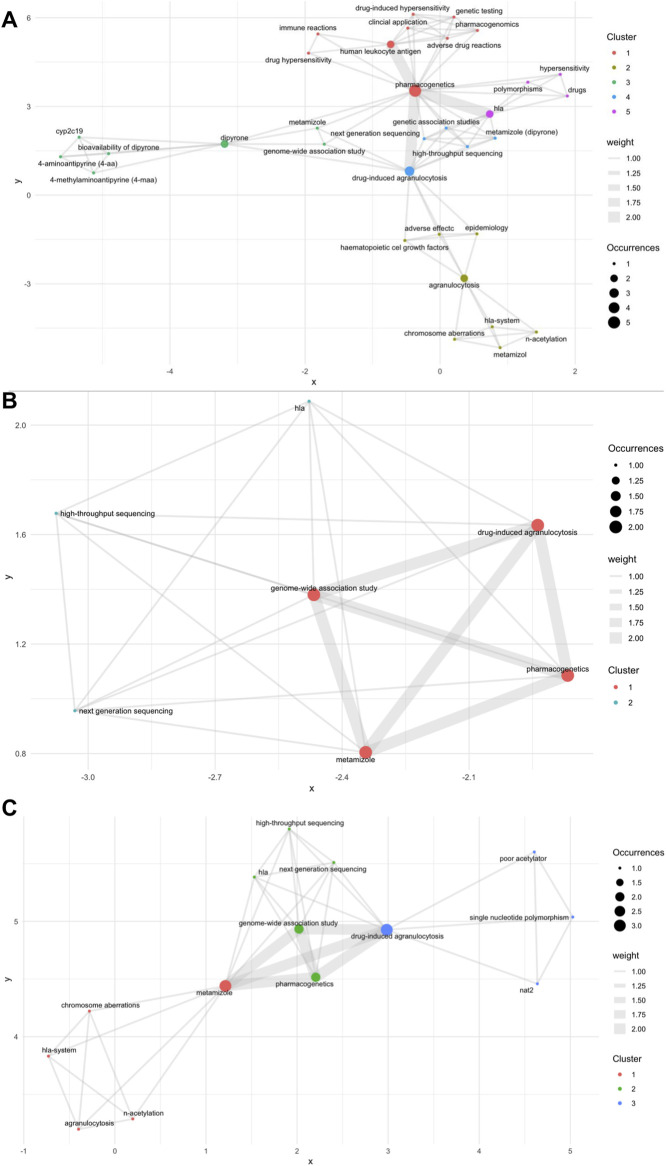
Bibliometric analysis; **(A)** Scopus database; **(B)** PubMed database; **(C)** Web of Science database. Nodes represent specific keywords; the size of each node reflects the frequency of occurrence. Edges (lines) indicate co-occurrence relationships between keywords; the thickness of the line denotes the strength of the connection (weight). Clusters (node colors: red, green, blue) represent groups of keywords that tend to co-occur, as determined by modularity optimization in the VOSviewer algorithm. Axes (x, y) are dimensionless and represent spatial proximity in the network based on similarity of keyword occurrence, not real-world coordinates. Distance between nodes indicates the strength of association: the closer two nodes, the more often those keywords co-occur in the analyzed publications.

We conducted a bibliometric analysis across three major databases. Specifically, we examined Scopus, which included 16 articles; PubMed, which contained four articles; and Web of Science, which comprised seven articles (Figure 2). This analysis illustrates the limited number and fragmentation of studies on the topic, highlighting the scarcity of comprehensive research within the existing literature. In the bibliometric visualization of the three databases, two genes, *HLA* and *NAT*, were identified. Notably, *HLA* was found in both the Scopus and Web of Science databases, whereas *NAT* was exclusively identified in the Scopus database. Despite these findings, it is challenging to draw definitive conclusions regarding the relationship between the identified genes and agranulocytosis. The limited number of articles available imposes significant constraints on the visualization and interpretation of these relationships.

### Associations between MIA and genetic variants

The findings of the four studies included are described in Table 3. The studies reported data investigating *SVEP1* (Sushi, Von Willebrand Factor Type A, EGF And Pentraxin Domain Containing 1) (Cismaru A. et al., 2020), *HLA* (Major Histocompatibility Complex) (Vlahov et al., 1996; Cismaru A. L. et al., 2020), *NAT2* (N-Acetyltransferase 2) (Radulovic et al., 2021), *CYP2C9* (Cytochrome P450 family two subfamily C member 9) and *CYP2C19* (Cytochrome P450 family two subfamily C member 19) (Radulovic et al., 2021) genes related to MIA.

**TABLE 3:** Genes studied in this systematic review.

Author, year	Sample	Study design	Genotype method	Gene	dbID/rs (genetic variants/Polymorphism)	Population	Allele frequency (%)		RR	OR	95%CI	*p-value*
							Cases	Controls				
Vlahov/1996 (Vlahov et al., 1996)	Blood	Retrospective (CC)	PCR-SSO	*HLA*	A2	Bulgaria	NR	NR	0.20	NA	NA	NR
					A24				25.20			**= 0.05**
					B7				9.30			NR
					DQw1				14.60			**= 0.05**
					DQA1*05:01				0.06			**= 0.05**
Cismaru/2020a (Cismaru et al., 2020a)	Blood	Retrospective (CC)	Genome-Wide SNP Arrays	*HLA*	B*35:01	Switzerland	0	7		0	0–0.86	0.022
						Germany	0	7	NA	0	0–2.8	0.37
						Spain	7	3		2.1	0.46–7.5	0.26
					C*04:01	Switzerland	0	14		0	0–0.42	**0.00042**
						Germany	4	9	NA	0.46	0.010–3.2	0.70
						Spain	14	17		0.77	0.30–1.8	0.70
					C*07:04	Switzerland	7	1		5.4	1.0–26	0.023
						Germany	0	3	NA	0	0–8.6	1.0
						Spain	2	1		2.5	0.042–49	0.42
					DQA1*01:02	Switzerland	25	19	NA	1.4	0.69–2.7	0.30
						Germany	29	19		1.7	0.57–4.9	0.28
						Spain	21	17		1.3	0.58–2.7	0.46
					DQB1*05:01	Switzerland	7	11		0.56	0.14–1.6	0.37
						Germany	8	12	NA	0.67	0.072–3.1	1.0
						Spain	21	13		1.7	0.76–3.7	0.15
					DQB1*06:04	Switzerland	13	5		2.8	1.0–7.0	0.039
						Germany	4	5	NA	0.76	0.017–5.8	1.0
						Spain	3	2		1.4	0.14–7.8	0.65
					DRB1*13:02	Switzerland	13	6		2.5	0.92–6.2	0.048
						Germany	4	5	NA	0.76	0.017–5.8	1.0
						Spain	3	3		1.0	0.10–4.9	1.0
					DRB1*04:01	Switzerland	13	5		3.1	1.1–8.0	0.015
						Germany	12	8	NA	1.7	0.29–7.0	0.42
						Spain	0	2		0	0–3.5	0.61
Cismaru/2020b (Cismaru et al., 2020b)	Blood	Retrospective (CC)	Genome-Wide SNP Arrays	-	rs55898176	Switzerland	0.27	0.065	NA	4.01	2.41–6.68	**1.01 × 10** ^**−7**^
						Germany	0.25	0.081				
						Spain	0.24	0.12				
				*SVEP1*	rs4427239	Switzerland	0.16	0.029	NA	5.47	2.81–10.65	**5.75 × 10** ^**−7**^
						Germany	0.041	0.016				
						Spain	0.15	0.041				
				*-*	rs112223975	Switzerland	0.27	0.065	NA	3.89	2.34–6.48	1.50 × 10^−7^
						Spain	0.24	0.12				
						Germany	0.25	0.081				
				*-*	rs11790418	Switzerland	0.27	0.065	NA	3.81	2.29–6.35	2.54 × 10^−7^
						Germany	0.25	0.098				
						Spain	0.24	0.12				
				*-*	rs1434481	Switzerland	0.27	0.065	NA	3.81	2.29–6.35	2.54 × 10^−7^
						Germany	0.25	0.098				
						Spain	0.24	0.11				
				*-*	rs28475568	Switzerland	0.27	0.065	NA	3.81	2.29–6.35	2.54 × 10^−7^
						Germany	0.25	0.098				
						Spain	0.24	0.11				
				*-*	rs28649995	Switzerland	0.27	0.065	NA	3.81	2.29–6.35	2.54 × 10^−7^
						Germany	0.25	0.098				
						Spain	0.24	0.11				
				*-*	rs56285046	Switzerland	0.28	0.073	NA	3.70	2.27–6.05	3.25 × 10^−7^
						Germany	0.25	0.092				
						Spain	0.24	0.12				
				*-*	rs77949268	Switzerland	0.27	0.078	NA	3.59	2.20–5.87	3.74 × 10^−7^
						Germany	0.25	0.10				
						Spain	0.26	0.12				
				*-*	rs10759436	Switzerland	0.15	0.029	NA	5.81	2.92–11.54	8.13 × 10^−7^
						Germany	0.041	0.016				
						Spain	0.15	0.041				
Radulovic/2021 (Radulovic et al., 2021)	NR	Retrospective (C)	Sanger sequencing	*NAT2*	*5, *6, *7, *11, *12, *13 and *14	Germany	NR	NR	NA	NR	NR	NR
				*CYP2C9*	*2, *3, *4 and 5*							
				*CYP2C19*	*2, *3 and *17							

Abbreviations: PCR-SSO, Polymerase chain reaction sequence-specific oligonucleotide; NR, not reported; NA, not applicable; OR, odds ratio; *SVEP1*, sushi, Von Willebrand Factor Type A, EGF, And Pentraxin Domain Containing 1; *HLA*, major histocompatibility complex; *NAT2*, N-Acetyltransferase 2; *CYP2C9*, Cytochrome P450 family two subfamily C member 9; *CYP2C19*, Cytochrome P450 family two subfamily C member 19; significant results in bold.

Most studies used blood samples for genotyping (Vlahov et al., 1996; Cismaru A. et al., 2020; Cismaru et al., 2020); however, one study did not report the specific type of biological material employed (Radulovic et al., 2021). Two of the four studies included used genome-wide arrays as the genotyping method (Cismaru A. et al., 2020; Cismaru et al., 2020), one used Sanger sequencing (Radulovic et al., 2021) and one used PCR-SSO (Vlahov et al., 1996).

Significant associations were observed exclusively with genetic variants in the *SVEP1* and HLA genes. In the HLA gene, associations were identified with the A24, DQw1, DQA1*05:01 and C*04:01 variants (Vlahov et al., 1996). In the chromosome nine region, associations were identified with the rs55898176 and rs4427239 variants, the latter of which is situated in the *SVEP1* gene (Cismaru A. et al., 2020). Two articles (Vlahov et al., 1996; Cismaru A. L. et al., 2020) investigated the HLA gene; the remaining genes were cited only in one article. Cismaru 2020b analyzed the association between HLA alleles and MIA among a Swiss, a Spanish and a German cohort. Only HLA-C*04:01 showed to be significantly associated with MIA in the Swiss cohort, but not in the Spanish and German cohorts (Cismaru A. L. et al., 2020).

A meta-analysis could not be performed due to the insufficient number of studies that investigated the association between genetic variants and agranulocytosis, as well as the considerable heterogeneity in the reporting of data in the available studies. However, data from each study could be analyzed individually.

### Frequency of genetic variants in different genetic ancestry groups

To investigate the influence of genetic variants, we conducted a literature review to ascertain allele frequencies in different genetic ancestry groups. To this end, our analysis focused exclusively on the genetic variants assessed by the case-control studies and evaluated the legal status of metamizole in the countries, to establish a comparison.

The legal status of metamizole can be classified into three general categories: withdrawn (WD), per prescription (PP), and over the counter (OTC). In certain countries, including the United States, Sweden, Finland, England, and Japan, the use of metamizole is prohibited. In Italy, Germany, Portugal, and Spain, metamizole can only be obtained per prescription. In several Latin American (such as Brazil, Argentina, and Mexico) and African (Cameroon and Egypt) countries, the use of metamizole is allowed, classified as over the counter (see Supplementary Table S4 for details).

### Frequencies of rs4427239 and rs55898176 across genetic ancestry groups

**FIGURE 3:**
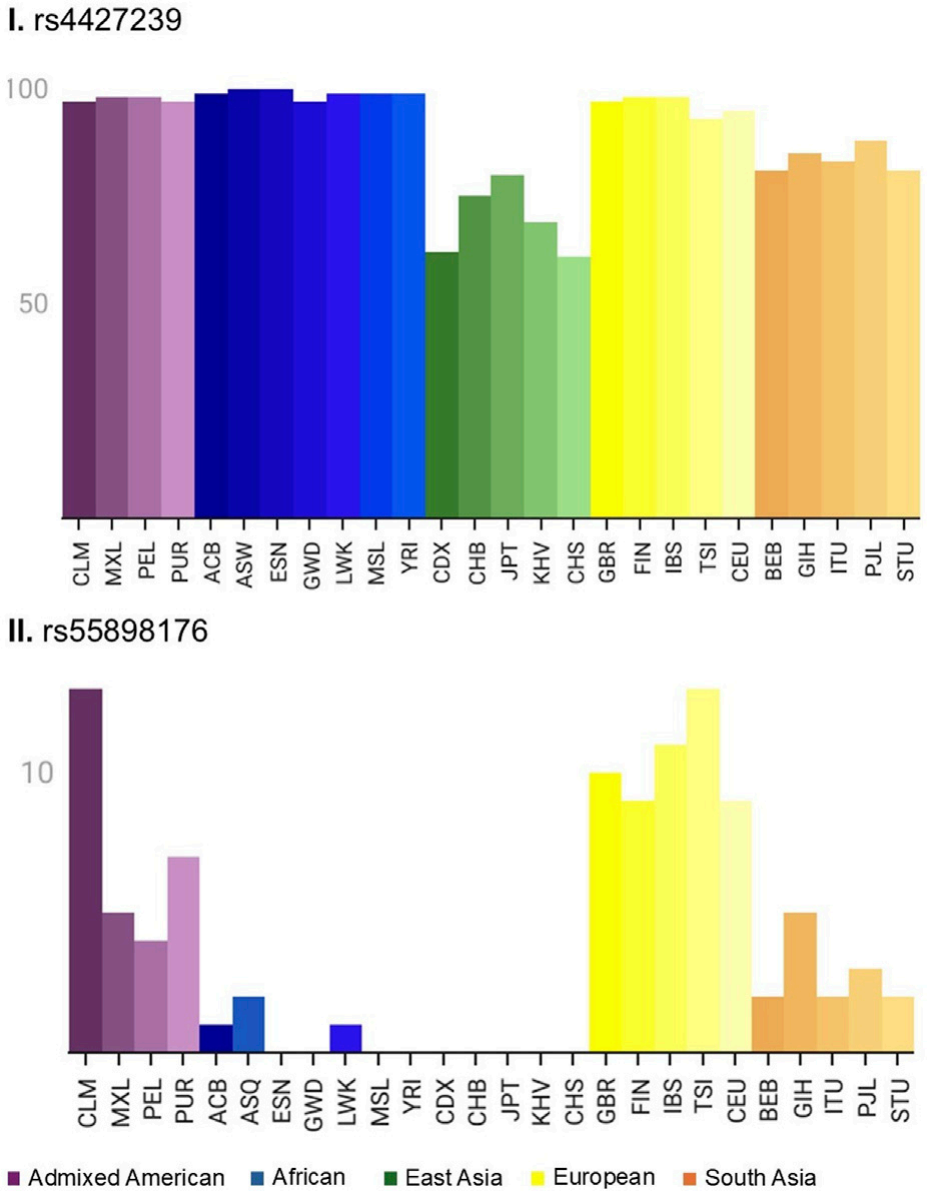
Global distribution of rs4427239 (I) and rs55898176 (II). Bar plots represent the frequency of the reference allele for each variant based on continental and subcontinental population groups. Colors indicate major continental ancestries (Purple: Americas; Blue: Africa; Green: East Asian; Yellow: Europe; Orange: South Asian), with shades distinguishing subpopulations as defined in the gnomAD v4.1.0 (https://gnomad.broadinstitute.org/). CLM, Colombians from Medellin; Colombia; MXL, Mexican Ancestry from Los Angeles; USA; PEL, Peruvians from Lima; Peru; PUR, Puerto Ricans from Puerto Rico; ACB, African Caribbeans in Barbados; ASQ, Americans of African Ancestry in SW; USA; ESN, Esan in Nigeria; GWD, Gambian in Western Divisions in the Gambia; LWK, Luhya in Webuye; Kenya; MSL, Mende in Sierra Leone; YRI, Yoruba in Ibadan; Nigeria; CDX, Chinese Dai in Xishuangbanna; China; CHB, Han Chinese in Beijing; China; JPT, Japanese in Tokyo; Japan; KHV, Kinh in Ho Chi Minh City; Vietnam; CHS, Southern Han Chinese; GBR, British in England and Scotland; FIN, Finnish in Finland; IBS, Iberian Population in Spain; TSI, Toscani in Italia; CEU, Utah Residents (CEPH) with Northern and Western European Ancestry; BEB, Bengali from Bangladesh; GIH, Gujarati Indian from Houston; Texas; ITU, Indian Telugu from the UK; PJL, Punjabi from Lahore; Pakistan; STU, Sri Lankan Tamil from the UK. Abbreviations, CLM, Colombians from Medellin; Colombia; MXL, Mexican Ancestry from Los Angeles; USA; PEL, Peruvians from Lima; Peru; PUR, Puerto Ricans from Puerto Rico; ACB, African Caribbeans in Barbados; ASQ, Americans of African Ancestry in SW; USA; ESN, Esan in Nigeria; GWD, Gambian in Western Divisions in the Gambia; LWK, Luhya in Webuye; Kenya; MSL, Mende in Sierra Leone; YRI, Yoruba in Ibadan; Nigeria; CDX, Chinese Dai in Xishuangbanna; China; CHB, Han Chinese in Beijing; China; JPT, Japanese in Tokyo; Japan; KHV, Kinh in Ho Chi Minh City; Vietnam; CHS, Southern Han Chinese; GBR, British in England and Scotland; FIN, Finnish in Finland; IBS, Iberian Population in Spain; TSI, Toscani in Italia; CEU, Utah Residents (CEPH) with Northern and Western European Ancestry; BEB, Bengali from Bangladesh; GIH, Gujarati Indian from Houston; Texas; ITU, Indian Telugu from the UK; PJL, Punjabi from Lahore; Pakistan; STU, Sri Lankan Tamil from the UK.

The allele frequencies of rs4427239 and rs55898176 were analyzed across five different genetic ancestry groups (Admixed American, African, East Asian, European, and South Asian) (Figure 3). rs4427239 exhibited an allele frequency variation ranging from 81% to 100% among all the subgroups. Despite their similar frequencies, especially among African subgroups, a distinct regulatory aspect of metamizole was observed. In contrast, rs55898176 was rare in East Asian populations, while European subpopulations exhibited a substantially higher prevalence, with frequencies ranging from 9% to 13% (see Supplementarys Tables S5 and S6 for details).

### Geographic distribution of HLA allele frequencies

**FIGURE 4:**
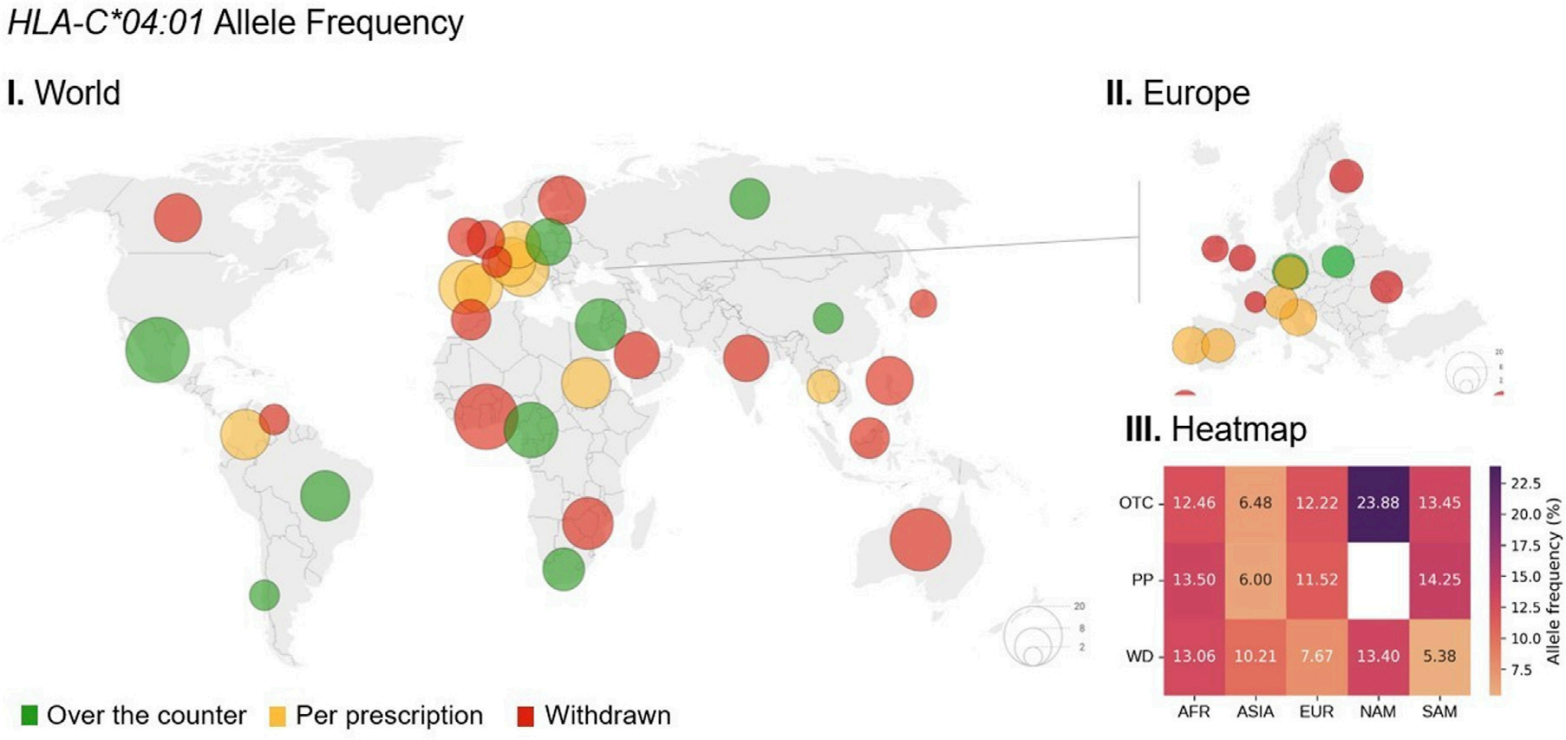
Global distribution of HLA-C*04:01 allele frequencies in relation to metamizole legal status. I. World map illustrating the frequency of the HLA-C*04:01 allele across different countries. The size of each circle is proportional to the allele frequency in that region, with the largest circles corresponding of a frequency of 20%. Circle colors indicate the legal status of metamizole in each country: green for over the counter (OTC), yellow for prescription-only (PP), and red for withdrawn (WD). II. Regional map of Europe showing HLA-C*04:01 allele frequencies and metamizole legal status. III. Heatmap displaying the average allele frequency (%) stratified by continent—Africa (AFR), Asia (ASIA), Europe (EUR), North America (NAM), and South America (SAM)—and metamizole legal status. Data from Allele Frequency Net Database (AFND) (https://www.allelefrequencies.net/default.asp).

The geographic distribution of the significant *HLA* alleles frequency (C*04:01) was analyzed in parallel with the legal status of metamizole in continents in Figure 4 (see Supplementary Figures S1–S7; Supplementary Tables S7–S14 for details). All continents had a sample size greater than 1,000 individuals, except Oceania, for alleles HLA-B*35:01 (*n* = 428) and HLA-DQA1*01:02 (*n =* 804) (see Supplementary Tables S7–S14 for additional details).

*HLA* alleles show varying frequencies among continents, regardless of metamizole legal status (Figure 3; Supplementary Figures S1–S7). The C*04:01 allele was more prevalent in North America, with a frequency ranging from 12.9% in Canada (*n =* 59) to 23.9% in Mexico (*n =* 1,133). Conversely, lower frequencies were observed in Asia, ranging from 4.4% in Japan (*n =* 20,835) to 15.2% in Israel (*n =* 74,942) (Supplementary Table S14).

When considering metamizole legal status, the frequency of *HLA* alleles seems generally similar among countries with different regulatory classifications. Despite a significant association being found for C*04:01 in one of the studies included, the similarity in the frequencies of this allele across countries is particularly evident, as African countries exhibit very similar frequencies despite differing regulatory statuses. A similar pattern is observed in European countries. The most notable contrast for this allele was observed in South America, where WD-classified countries showed distinct frequencies compared to others (OTC and PP). Among all *HLA*-alleles, only DRB1*13:02 seemed to show consistently higher allele frequencies in countries with WD status compared with countries with OTC or PP status among all continents (Supplementary Figure S4). However, this allele did not show any significant association in the studies included in this review.

In countries where metamizole is classified as OTC, no consistent pattern of significantly higher or lower allele frequencies was observed compared to countries with some level of restriction (PP or WD). The frequency of C*04:01 ranged from 6.48% (*n =* 16,143) in Asia to 23.88% in North America (*n =* 1,133) among OTC-classified countries. A similar trend was observed for B*35:01 and in WD-classified countries, particularly for the alleles DQA1*01:02, DQB1*05:01, and DRB1*04:01. Overall, no consistent patterns were identified that could explain the different legal statuses of metamizole across countries.

## Discussion

To the best of our knowledge, this is the first systematic review to investigate the association between genetic variants and MIA. Of the four studies included in the review, three were case-control studies (Vlahov et al., 1996; Cismaru A. L. et al., 2020; Cismaru et al., 2020) and one was a case report (Radulovic et al., 2021), showing a surprisingly small number of studies. Furthermore, the quality classification ranged from four to seven stars, and notably, none scored on the “comparability” criterion. This indicates that none of them reported how the cohort of exposed and unexposed individuals was compared, indicating a potential bias. A key limitation resulting from this is the lack of control for potential confounding factors, such as concomitant medications and comorbidities, which may have influenced the reported associations and reduced the internal validity of the findings. Despite these limitations, none of the studies were classified as low quality using the NOS criteria.

### Studies included

The association between *HLA* variants and MIA was investigated in two studies: Vlahov et al. (1996) and Cismaru A. et al. (2020). Vlahov et al. (1996) investigated the *HLA* gene among nine patients with MIA in Sofia (Bulgaria). Although the presence of concomitant medications was not detailed, it states that MIA cases met International Agranulocytosis and Aplastic Anemia Study (IAAAS) requirements. The A24, B7, DQw1, and DQA1*05:01 antigens demonstrated statistical significance with MIA through relative risk assessment. Furthermore, *HLA-2* antigen seems to play a protective role against MIA in the Bulgarian population. According to the authors, there was cytogenetic evidence that hereditary factors in the etiology of agranulocytosis could be associated with more than one gene. However, this study evaluated only nine patients with agranulocytosis, which limits the generalizability of the findings. Therefore, these associations should be interpreted with caution.

Conversely, the study conducted by Cismaru A. L. et al. (2020) revealed no statistically significant correlations between HLA alleles and MIA in European populations from Switzerland, Germany, and Spain, evaluated from 2005 to 2017. Although the study identified individual associations in eight candidate alleles across five distinct classical class I and II genes (B*35:01, C*04:01, C*07:04, DQA1*01:02, DQB1*05:01, DQB1*06:04, DRB1*13:02, and DRB1*04:01), these were not corroborated when the data were evaluated with all three cohorts. Only C*04:01 showed significance in the Swiss cohort, with no replication in the others. Despite the absence of significant results, the authors state that the study had sufficient power to detect associations between rarer and more common alleles with clinically relevant frequency effect sizes (Cismaru A. L. et al., 2020).

The studies by Cismaru (Cismaru A. et al., 2020; Cismaru et al., 2020) correspond to the same cohort, and applied genome-wide genotyping, high-throughput resequencing (HTS), and high-resolution typing of eight HLA loci. The second genome-wide association study (GWAS) by Cismaru A. et al. (2020) identified significant associations between MIA and genetic variants in European populations from Switzerland, Germany, and Spain. Two candidate loci on chromosome nine were identified: rs55898176 and rs4427239. The first corresponds to a SNP near the long non-coding RNA of the *CAAP1* gene and the genetic variant rs4427239 (chr9p13) is located in the *SVEP1* gene. The *CAAP1* gene has been identified as a negative regulator of the intrinsic apoptosis pathway, influencing caspase expression and activity (Samuelov et al., 2017; Zhang et al., 2020). Additionally, the *SVEP1* gene has been shown to play a role in epidermal development and keratinocyte differentiation, independent of cell-cell adhesion (Samuelov et al., 2017). A total of 84 candidate genes were analyzed in the Swiss cohort, with replication of findings and a genome-wide meta-analysis conducted in two other independent cohorts (Germany and Spain), and large cohort of participants, supporting the robustness of the findings (Cismaru A. et al., 2020). Nevertheless, the study grouped cases of MIA and MIN and did not discuss the potential influence of concomitant medications, even though information on antibiotics, analgesics, and beta-lactams was retrieved from medical records.

A key limitation of the studies by Cismaru was the unclear criteria for establishing causality between metamizole and agranulocytosis. Although cases were selected to minimize confounding, the lack of standardized causality assessment and limited discussion of co-medications may have impacted the reliability of the findings. Another important limitation is the strategy of pooling healthy and unrelated individuals with metamizole-tolerant patients (i.e., those who had received at least 500 mg of metamizole per day for a minimum of 28 consecutive days) in the control group. This approach may introduce a bias into the analysis, as tolerant patients might carry protective genetic factors. The resulting heterogeneity within the control group could reduce the statistical power to detect risk alleles associated with MIA, ultimately limiting the validity and generalizability of the associations found.

Variants in drug-metabolizing enzymes have been investigated by Radulovic et al. (2021). Radulovic et al. (2021) is a case series that evaluated three patients who developed severe agranulocytosis after metamizole intake. Genotyping was conducted for *NAT2*, CYP2C9*, *3, *4 and 5, CYP2C19*2, *3 and *17, and NAT2*5*, *6, *7, *11, *12, *13, and *14. The phenotypes associated with intermediate metabolizers (*CYP2C19*(*1/*2)), poor metabolizers (*CYP2C9*(*3/*3)), and slow acetylators (*NAT2*(*4/*5U)) are likely to result in the accumulation of toxic metabolites, thereby inducing severe agranulocytosis, as the study indicates. As it is a case series, the data are not robust enough to prove the results in a large population (Radulovic et al., 2021).

Therefore, significant results were only observed in the studies conducted by Cismaru A. L. et al. (2020) and Vlahov et al. (1996), regarding the *HLA* and *SVEP1* genes, respectively. This limits the ability to conduct a comprehensive analysis or even a meta-analysis between existing studies (Vlahov et al., 1996; Cismaru A. et al., 2020; Cismaru et al., 2020; Radulovic et al., 2021).

### Frequency of genetic variants

As indicated by the European Medicines Agency (EMA), the potential for agranulocytosis induction may be linked to the genetic attributes of the studied population (EMA, 2019). Therefore, this study examined the frequency of significant genetic variants (rs55898176 and rs4427239 located on chromosome nine and HLA-C*04:01) identified in the systematic review across diverse populations, alongside an analysis of metamizole’s legal status.

Importantly, the significant associations found were derived from European cohorts (Switzerland, Germany, and Spain). In addition, the association for C*04:01 was detected only when analyzed individually in the Swiss cohort, further restricting the scope of the findings to a specific region. This limitation restricts the extrapolation of findings to other populations, particularly those with a high degree of admixture.

The data obtained reveal considerable variation in allele frequencies, even among countries with the same legal status. For most alleles, no pattern was identified that could explain regulatory differences, such as a correlation between restricted use and higher or lower frequencies compared to other countries. Even among OTC-classified countries, the frequency of C*04:01 varied widely between Asian and North American populations, for example,. Similarly, in European countries - the focus of studies reporting significant associations - C*04:01 allele frequencies remained consistent across different legal classifications.

Some alleles exhibited contrasts between OTC- and WD-classified countries, warranting further investigation into their potential influence. The DRB1*13:02 allele stands out in this context. In Africa, the alleles DQB1*05:01, DRB1*04:01, and DRB1*13:02 showed notable differences, while in North America, B*35:01 and C*04:01 were particularly prominent.

Although significant associations were found between MIA and the variants discussed in the systematic review, their frequencies do not justify the differing regulations of metamizole observed worldwide. This result suggests that genetic factors may not be determinant in the regulation of metamizole across different countries, making the hypothesis that agranulocytosis is linked to genetic variants appear arbitrary. These findings highlight the need for further studies to investigate a potential correlation between allele frequency and the development of MIA in these specific populations, with particular focus on Africa, which remains significantly underrepresented in studies on HLA variation (Pagkrati et al., 2023).

In general, the data on each HLA allele remains controversial. HLA alleles have been linked to hypersensitivity reactions to various medications (Shah, 2019), and their correlation with drug-related adverse reactions are well documented in scientific literature. This association has been particularly well-studied regarding carbimazole-induced agranulocytosis (Cheung et al., 2016; Fan et al., 2017; Chen and Chi, 2019; Konte et al., 2021), but further research is needed for MIA. As stated by Fan et al. (2017), most ADRs associated with HLA are ethnically specific (Fan et al., 2017). It is, therefore, crucial to ensure that studies conducted in specific locations are not extrapolated to diverse populations to prevent misrepresentations.

### Agranulocytosis and safety of metamizole

Currently, countries have banned or withdrawn metamizole from the market based on studies that reported the risk of MIA. One such study, which has gained international recognition, was conducted in Sweden and involved 14 documented cases of agranulocytosis from 1995 to 1999—yielding an incidence of one case per 1,493 prescriptions. Notwithstanding the high incidence reported, the published studies generally had a small number of samples and limitations that may have introduced bias into the results (Shah, 2019), such as a failure to distinguish between neutropenia, agranulocytosis, and aplastic anemia (Eleutério et al., 2024).

Despite restrictions in different countries, metamizole remains one of the most widely used medications in Latin America and other regions, often available without a prescription (van der Klauw et al., 1999; Hamerschlak et al., 2008; Basak et al., 2010; Blaser et al., 2015; Maciá-Martínez et al., 2024). Maciá-Martínez et al. (2024) reported an incidence of MIA in Spain - a PP-classified country - of 0.85 cases per million person-weeks, using data collected between 2005 and 2022 (Maciá-Martínez et al., 2024). In contrast, the Latin American case-control study conducted in Brazil, Argentina, and Mexico - where metamizole is OTC - reported an overall incidence rate of agranulocytosis of 0.38 cases per million inhabitant-years, between 2002 and 2005 (Hamerschlak et al., 2008). These findings suggest that the incidence of MIA is not necessarily higher in countries where metamizole is OTC. However, direct comparisons are limited due to differences in study design, time frame, and population characteristics.

Moreover, accurately assessing MIA remains challenging. The unknown time required for agranulocytosis induction, along with the variable interval between symptom onset, diagnosis and reporting timelines, complicates surveillance efforts (Maciá-Martínez et al., 2024). Despite these uncertainties, available evidence suggests that the risk of developing agranulocytosis increases with prolonged use and typically resolves within 10 days after discontinuation of metamizole (Ibáñez et al., 2005). Given its widespread use and the relatively low incidence of MIA, especially in OTC-classified countries, these findings further support the drug’s safety profile.

An element reinforcing the safety of metamizole use is its comparison to other analgesics. According to Eleutério et al. (2024), when compared to ibuprofen and acetylsalicylic acid for the treatment of mild to moderate pain, metamizole, at any dose, demonstrated a 38.8% lower likelihood of causing adverse effects compared to acetaminophen and acetylsalicylic acid (Eleutério et al., 2024). Additionally, in comparing metamizole to paracetamol, no significant differences in ADRs were observed, and short-term exposure mortality rates were similar (Kötter et al., 2015). For Maj and Lis (2002), the evidence suggests that metamizole offers a comparable, if not superior, risk-benefit profile relative to other drugs outside the scope of pharmacopolitical debate (Maj and Lis, 2002).

Despite its safety profile, it is not possible to rule out that environmental factors and patterns of drug use, such as higher doses or longer exposure periods, may contribute to variations in incidence rates (Ibáñez et al., 2005; Hamerschlak et al., 2008). Further studies are essential to deepen the understanding of metamizole’s safety across different usage conditions.

Although this systematic review contributes to a better understanding of potential pharmacogenetic risk factors for MIA and highlights regulatory discrepancies worldwide, several limitations must be acknowledged. First, the small number of eligible studies represents a major limitation. This can be attributed to several factors. Although agranulocytosis is a well-known adverse event associated with metamizole, it remains a rare condition (Akyay and Deveci, 2015), which limits the availability of studies involving a sufficiently large number of cases. Most of the existing studies are based on spontaneous adverse event reports, which are useful for hypothesis generation but provide limited epidemiological evidence (Carvalho et al., 2023). Additionally, the use of metamizole is banned in several countries (Hamerschlak et al., 2008; Basak et al., 2010; Shah, 2019; Guimarães et al., 2021; Eleutério et al., 2024), further reducing the availability of relevant data across populations. However, our search strategy, although rigorous, was as broad as possible to include all studies addressing the pharmacogenetics of MIA, highlighting the scarcity of research in this field.

Second, the statistical power of the studies retrieved is compromised due to very small sample sizes. This likely reflects both the rarity of MIA and the challenges in establishing a clear causal relationship between agranulocytosis events and metamizole use. Therefore, the findings of these studies should be interpreted with caution. Additional studies are warranted to confirm these genetic associations.

Third, the heterogeneity among the included studies also posed challenges. The studies varied considerably in terms of populations studied, criteria to attribute causality to metamizole, and the specific genetic variants investigated. While all studies used a similar hematological threshold to define agranulocytosis (ANC <0.5 × 10^9^/L), the methods for attributing causality to metamizole, such as co-medication, were inconsistently reported. These methodological limitations may have influenced the internal validity of the associations reported. Due to these factors, a meta-analysis was not feasible, as pooling heterogenous data could have resulted in misleading conclusions. Instead, we opted for a narrative synthesis, which allowed for a more nuanced interpretation of the evidence.

Fourth, despite efforts to comprehensively search the literature, the review was restricted to studies published in English or Spanish, which may have introduced language bias. Additionally, publication bias may have occurred, as studies with positive findings are more likely to be published. Furthermore, we did not perform backward and forward citation tracking, which may have reduced the number of articles retrieved. Instead, we opted to rely on a structured database search strategy to ensure reproducibility and transparency. Despite these limitations, this review highlights the current gap in the literature and underscores the urgent need for well-designed, multicenter pharmacogenetic studies to clarify the genetic susceptibility to MIA and inform regulatory decisions.

## Conclusion

This systematic review included four studies in total. Among these, only two reported significant associations with MIA, specifically HLA-C*04:01 and variants in the chromosomal region 9 (rs55898176 and rs4427239). However, due to the limited data available in the literature, it was not possible to perform comparative analyses to validate these associations. Although allele frequencies were analyzed in populations with varying regulatory statuses of metamizole, the findings were inconsistent. There is insufficient evidence to justify its prohibition in certain countries based solely on genetic variants. Consequently, further research is needed to evaluate the impact of metamizole use and the prevalence of MIA in different populations in relation to regulatory frameworks. Moreover, given the critical role of the *HLA* gene in drug-induced adverse reactions, additional studies are warranted to better characterize populations and investigate potential genetic associations, particularly concerning HLA-C*04:01 allele.

## Data Availability

The original contributions presented in the study are included in the article/Supplementary Materials, further inquiries can be directed to the corresponding author.
