# Supplementary material for: Pharmacogenetics of metamizole-induced agranulocytosis: a systematic review and drug regulation implications
Source: Front Pharmacol. 2025 Sep 24;16:1624044. doi: 10.3389/fphar.2025.1624044 (PMC12504837; doi:10.3389/fphar.2025.1624044)
Supplement: Supplementary file 1 [file DataSheet1.docx]

Supplementary Material

1. **Supplementary Tables**

**Table S1.** PRISMA checklist.

| **Section and Topic** | **Item #** | **Checklist item** | **Location where item is reported** |
| --- | --- | --- | --- |
| **TITLE** | | |  |
| Title | 1 | Identify the report as a systematic review. | Title |
| **ABSTRACT** | | |  |
| Abstract | 2 | See the PRISMA 2020 for Abstracts checklist. | - |
| **INTRODUCTION** | | |  |
| Rationale | 3 | Describe the rationale for the review in the context of existing knowledge. | Introduction |
| Objectives | 4 | Provide an explicit statement of the objective(s) or question(s) the review addresses. | Introduction |
| **METHODS** | | |  |
| Eligibility criteria | 5 | Specify the inclusion and exclusion criteria for the review and how studies were grouped for the syntheses. | Materials and Methods |
| Information sources | 6 | Specify all databases, registers, websites, organisations, reference lists and other sources searched or consulted to identify studies. Specify the date when each source was last searched or consulted. | Materials and Methods |
| Search strategy | 7 | Present the full search strategies for all databases, registers and websites, including any filters and limits used. | Materials and Methods |
| Selection process | 8 | Specify the methods used to decide whether a study met the inclusion criteria of the review, including how many reviewers screened each record and each report retrieved, whether they worked independently, and if applicable, details of automation tools used in the process. | Materials and Methods |
| Data collection process | 9 | Specify the methods used to collect data from reports, including how many reviewers collected data from each report, whether they worked independently, any processes for obtaining or confirming data from study investigators, and if applicable, details of automation tools used in the process. | Materials and Methods |
| Data items | 10a | List and define all outcomes for which data were sought. Specify whether all results that were compatible with each outcome domain in each study were sought (e.g. for all measures, time points, analyses), and if not, the methods used to decide which results to collect. | Materials and Methods |
|  | 10b | List and define all other variables for which data were sought (e.g. participant and intervention characteristics, funding sources). Describe any assumptions made about any missing or unclear information. | Materials and Methods |
| Study risk of bias assessment | 11 | Specify the methods used to assess risk of bias in the included studies, including details of the tool(s) used, how many reviewers assessed each study and whether they worked independently, and if applicable, details of automation tools used in the process. | Materials and Methods |
| Effect measures | 12 | Specify for each outcome the effect measure(s) (e.g. risk ratio, mean difference) used in the synthesis or presentation of results. | Materials and Methods |
| Synthesis methods | 13a | Describe the processes used to decide which studies were eligible for each synthesis (e.g. tabulating the study intervention characteristics and comparing against the planned groups for each synthesis (item #5)). | Materials and Methods |
|  | 13b | Describe any methods required to prepare the data for presentation or synthesis, such as handling of missing summary statistics, or data conversions. | Materials and Methods |
|  | 13c | Describe any methods used to tabulate or visually display results of individual studies and syntheses. | Materials and Methods |
|  | 13d | Describe any methods used to synthesize results and provide a rationale for the choice(s). If meta-analysis was performed, describe the model(s), method(s) to identify the presence and extent of statistical heterogeneity, and software package(s) used. | Materials and Methods |
|  | 13e | Describe any methods used to explore possible causes of heterogeneity among study results (e.g. subgroup analysis, meta-regression). | Materials and Methods |
|  | 13f | Describe any sensitivity analyses conducted to assess robustness of the synthesized results. | Materials and Methods |
| Reporting bias assessment | 14 | Describe any methods used to assess risk of bias due to missing results in a synthesis (arising from reporting biases). | Materials and Methods |
| Certainty assessment | 15 | Describe any methods used to assess certainty (or confidence) in the body of evidence for an outcome. | Materials and Methods |
| **RESULTS** | | |  |
| Study selection | 16a | Describe the results of the search and selection process, from the number of records identified in the search to the number of studies included in the review, ideally using a flow diagram. | Results |
|  | 16b | Cite studies that might appear to meet the inclusion criteria, but which were excluded, and explain why they were excluded. | Results |
| Study characteristics | 17 | Cite each included study and present its characteristics. | Results |
| Risk of bias in studies | 18 | Present assessments of risk of bias for each included study. | Results |
| Results of individual studies | 19 | For all outcomes, present, for each study: (a) summary statistics for each group (where appropriate) and (b) an effect estimate and its precision (e.g. confidence/credible interval), ideally using structured tables or plots. | Results |
| Results of syntheses | 20a | For each synthesis, briefly summarise the characteristics and risk of bias among contributing studies. | Results |
|  | 20b | Present results of all statistical syntheses conducted. If meta-analysis was done, present for each the summary estimate and its precision (e.g. confidence/credible interval) and measures of statistical heterogeneity. If comparing groups, describe the direction of the effect. | Results |
|  | 20c | Present results of all investigations of possible causes of heterogeneity among study results. | Results |
|  | 20d | Present results of all sensitivity analyses conducted to assess the robustness of the synthesized results. | Results |
| Reporting biases | 21 | Present assessments of risk of bias due to missing results (arising from reporting biases) for each synthesis assessed. | Results |
| Certainty of evidence | 22 | Present assessments of certainty (or confidence) in the body of evidence for each outcome assessed. | Results |
| **DISCUSSION** | | |  |
| Discussion | 23a | Provide a general interpretation of the results in the context of other evidence. | Discussion |
|  | 23b | Discuss any limitations of the evidence included in the review. | Discussion |
|  | 23c | Discuss any limitations of the review processes used. | Discussion |
|  | 23d | Discuss implications of the results for practice, policy, and future research. | Discussion |
| **OTHER INFORMATION** | | |  |
| Registration and protocol | 24a | Provide registration information for the review, including register name and registration number, or state that the review was not registered. | Materials and Methods |
|  | 24b | Indicate where the review protocol can be accessed, or state that a protocol was not prepared. | Materials and Methods |
|  | 24c | Describe and explain any amendments to information provided at registration or in the protocol. | Materials and Methods |
| Support | 25 | Describe sources of financial or non-financial support for the review, and the role of the funders or sponsors in the review. | Funding |
| Competing interests | 26 | Declare any competing interests of review authors. | Conflict of Interest |
| Availability of data, code and other materials | 27 | Report which of the following are publicly available and where they can be found: template data collection forms; data extracted from included studies; data used for all analyses; analytic code; any other materials used in the review. | Materials and Methods |

*From:*  Page MJ, McKenzie JE, Bossuyt PM, Boutron I, Hoffmann TC, Mulrow CD, et al. The PRISMA 2020 statement: an updated guideline for reporting systematic reviews. BMJ 2021;372:n71. doi: 10.1136/bmj.n71

**Table S2.** Search Strategy

| **Databases** | **Search Strategy** | **April, 08, 2025** |
| --- | --- | --- |
| PUBMED | (((Dipyrone[MeSH Terms]) OR (Dipyrone[Title/Abstract] OR Methamizole[Title/Abstract] OR Metamizol[Title/Abstract] OR Dipyronium[Title/Abstract] OR Metamizole[Title/Abstract] OR Biopyrin[Title/Abstract] OR Novalgetol[Title/Abstract] OR Novalgin[Title/Abstract] OR Pyralgin[Title/Abstract] OR Novaminsulfone[Title/Abstract] OR Sulpyrin[Title/Abstract] OR Sulpyrine[Title/Abstract] OR Optalgin[Title/Abstract] OR "Noramidopyrine Methanesulfonate Sodium"[Title/Abstract] OR Novamidazophen[Title/Abstract] OR "Metamizole Sodium"[Title/Abstract] OR Methampyrone[Title/Abstract] OR Algopyrin[Title/Abstract] OR Analgin[Title/Abstract] OR Narone[Title/Abstract] OR "Noramidopyrine Methanesulfonate"[Title/Abstract])) AND (((((((Agranulocytosis[MeSH Terms]) OR (Agranulocytosis[Title/Abstract] OR Agranulocytoses[Title/Abstract] OR Granulocytopenia[Title/Abstract] OR Granulocytopenias[Title/Abstract])) OR (Granulopenia[Title/Abstract] OR panleukopenia[Title/Abstract])) OR ((Neutropenia[MeSH Terms]) OR (Neutropenia[Title/Abstract] OR Neutropenias[Title/Abstract]))) OR ("cyclic neutropaenia"[Title/Abstract] OR "cyclic neutropenia"[Title/Abstract] OR neutropaenia[Title/Abstract])) OR ((Cytopenia[MeSH Terms]) OR (Cytopenia[Title/Abstract] OR Cytopenias[Title/Abstract]))) OR (Cytopaenia[Title/Abstract]))) AND ((((((((((Polymorphism, Single Nucleotide[MeSH Terms]) OR ("Polymorphism, Single Nucleotide"[Title/Abstract] OR "Nucleotide Polymorphism, Single"[Title/Abstract] OR "Nucleotide Polymorphisms, Single"[Title/Abstract] OR "Polymorphisms, Single Nucleotide"[Title/Abstract] OR "Single Nucleotide Polymorphisms"[Title/Abstract] OR "SNPs"[Title/Abstract] OR "Single Nucleotide Polymorphism"[Title/Abstract])) OR ("single nucleotide variant"[Title/Abstract] OR "single nucleotide variation"[Title/Abstract])) OR ((Polymorphism, Genetic[MeSH Terms]) OR ("Polymorphism, Genetic"[Title/Abstract] OR "Polymorphisms, Genetic"[Title/Abstract] OR "Genetic Polymorphisms"[Title/Abstract] OR "Genetic Polymorphism"[Title/Abstract] OR "Polymorphism (Genetics)"[Title/Abstract] OR "Polymorphisms (Genetics)"[Title/Abstract]))) OR (Polymorphism[Title/Abstract] OR Polymorphisms[Title/Abstract])) OR ((Gene Frequency[MeSH Terms]) OR ("Gene Frequency"[Title/Abstract] OR "Frequencies, Gene"[Title/Abstract] OR "Frequency, Gene"[Title/Abstract] OR "Gene Frequencies"[Title/Abstract] OR "Allele Frequency"[Title/Abstract] OR "Allele Frequencies"[Title/Abstract] OR "Frequencies, Allele"[Title/Abstract] OR "Frequency, Allele"[Title/Abstract] OR "Genetic Equilibrium"[Title/Abstract] OR "Equilibrium, Genetic"[Title/Abstract]))) OR ("allelic frequency"[Title/Abstract])) OR ("gene variant"[Title/Abstract] OR "gene variants"[Title/Abstract] OR "genetic variants"[Title/Abstract] OR "genetic variant"[Title/Abstract])) OR ((Genetic Variation[MeSH Terms]) OR ("Genetic Variation"[Title/Abstract] OR "Genetic Variations"[Title/Abstract] OR "Variations, Genetic"[Title/Abstract] OR "Variation, Genetic"[Title/Abstract] OR "Diversity, Genetic"[Title/Abstract] OR "Diversities, Genetic"[Title/Abstract] OR "Genetic Diversities"[Title/Abstract] OR "Genetic Diversity"[Title/Abstract]))) OR ((Pharmacogenetics[MeSH Terms]) OR (Pharmacogenetics[Title/Abstract] OR Pharmacogenomics[Title/Abstract]))) | 04 |
| PUBMED PMC | (((Dipyrone[MeSH Terms]) OR (Dipyrone[Title/Abstract] OR Methamizole[Title/Abstract] OR Metamizol[Title/Abstract] OR Dipyronium[Title/Abstract] OR Metamizole[Title/Abstract] OR Biopyrin[Title/Abstract] OR Novalgetol[Title/Abstract] OR Novalgin[Title/Abstract] OR Pyralgin[Title/Abstract] OR Novaminsulfone[Title/Abstract] OR Sulpyrin[Title/Abstract] OR Sulpyrine[Title/Abstract] OR Optalgin[Title/Abstract] OR "Noramidopyrine Methanesulfonate Sodium"[Title/Abstract] OR Novamidazophen[Title/Abstract] OR "Metamizole Sodium"[Title/Abstract] OR Methampyrone[Title/Abstract] OR Algopyrin[Title/Abstract] OR Analgin[Title/Abstract] OR Narone[Title/Abstract] OR "Noramidopyrine Methanesulfonate"[Title/Abstract])) AND (((((((Agranulocytosis[MeSH Terms]) OR (Agranulocytosis[Title/Abstract] OR Agranulocytoses[Title/Abstract] OR Granulocytopenia[Title/Abstract] OR Granulocytopenias[Title/Abstract])) OR (Granulopenia[Title/Abstract] OR panleukopenia[Title/Abstract])) OR ((Neutropenia[MeSH Terms]) OR (Neutropenia[Title/Abstract] OR Neutropenias[Title/Abstract]))) OR ("cyclic neutropaenia"[Title/Abstract] OR "cyclic neutropenia"[Title/Abstract] OR neutropaenia[Title/Abstract])) OR ((Cytopenia[MeSH Terms]) OR (Cytopenia[Title/Abstract] OR Cytopenias[Title/Abstract]))) OR (Cytopaenia[Title/Abstract]))) AND ((((((((((Polymorphism, Single Nucleotide[MeSH Terms]) OR ("Polymorphism, Single Nucleotide"[Title/Abstract] OR "Nucleotide Polymorphism, Single"[Title/Abstract] OR "Nucleotide Polymorphisms, Single"[Title/Abstract] OR "Polymorphisms, Single Nucleotide"[Title/Abstract] OR "Single Nucleotide Polymorphisms"[Title/Abstract] OR "SNPs"[Title/Abstract] OR "Single Nucleotide Polymorphism"[Title/Abstract])) OR ("single nucleotide variant"[Title/Abstract] OR "single nucleotide variation"[Title/Abstract])) OR ((Polymorphism, Genetic[MeSH Terms]) OR ("Polymorphism, Genetic"[Title/Abstract] OR "Polymorphisms, Genetic"[Title/Abstract] OR "Genetic Polymorphisms"[Title/Abstract] OR "Genetic Polymorphism"[Title/Abstract] OR "Polymorphism (Genetics)"[Title/Abstract] OR "Polymorphisms (Genetics)"[Title/Abstract]))) OR (Polymorphism[Title/Abstract] OR Polymorphisms[Title/Abstract])) OR ((Gene Frequency[MeSH Terms]) OR ("Gene Frequency"[Title/Abstract] OR "Frequencies, Gene"[Title/Abstract] OR "Frequency, Gene"[Title/Abstract] OR "Gene Frequencies"[Title/Abstract] OR "Allele Frequency"[Title/Abstract] OR "Allele Frequencies"[Title/Abstract] OR "Frequencies, Allele"[Title/Abstract] OR "Frequency, Allele"[Title/Abstract] OR "Genetic Equilibrium"[Title/Abstract] OR "Equilibrium, Genetic"[Title/Abstract]))) OR ("allelic frequency"[Title/Abstract])) OR ("gene variant"[Title/Abstract] OR "gene variants"[Title/Abstract] OR "genetic variants"[Title/Abstract] OR "genetic variant"[Title/Abstract])) OR ((Genetic Variation[MeSH Terms]) OR ("Genetic Variation"[Title/Abstract] OR "Genetic Variations"[Title/Abstract] OR "Variations, Genetic"[Title/Abstract] OR "Variation, Genetic"[Title/Abstract] OR "Diversity, Genetic"[Title/Abstract] OR "Diversities, Genetic"[Title/Abstract] OR "Genetic Diversities"[Title/Abstract] OR "Genetic Diversity"[Title/Abstract]))) OR ((Pharmacogenetics[MeSH Terms]) OR (Pharmacogenetics[Title/Abstract] OR Pharmacogenomics[Title/Abstract]))) | 0 |
| BVS/BIREME | ((dipyrone OR methamizole OR metamizol OR dipyronium OR metamizole OR biopyrin OR novalgetol OR novalgin OR pyralgin OR novaminsulfone OR sulpyrin OR sulpyrine OR optalgin OR "Noramidopyrine Methanesulfonate Sodium" OR novamidazophen OR "Metamizole Sodium" OR methampyrone OR algopyrin OR analgin OR narone OR "Noramidopyrine Methanesulfonate")) AND ((agranulocytosis OR agranulocytoses OR granulocytopenia OR granulocytopenias) OR (granulopenia OR panleukopenia) OR (neutropenia OR neutropenias) OR ("cyclic neutropaenia" OR "cyclic neutropenia" OR neutropaenia) OR (cytopenia OR cytopenias) OR (cytopaenia)) AND (("Polymorphism, Single Nucleotide" OR "Nucleotide Polymorphism, Single" OR "Nucleotide Polymorphisms, Single" OR "Polymorphisms, Single Nucleotide" OR "Single Nucleotide Polymorphisms" OR "SNPs" OR "Single Nucleotide Polymorphism") OR ("single nucleotide variant" OR "single nucleotide variation") OR ("Polymorphism, Genetic" OR "Polymorphisms, Genetic" OR "Genetic Polymorphisms" OR "Genetic Polymorphism" OR "Polymorphism (Genetics)" OR "Polymorphisms (Genetics)") OR (polymorphism OR polymorphisms) OR ("Gene Frequency" OR "Frequencies, Gene" OR "Frequency, Gene" OR "Gene Frequencies" OR "Allele Frequency" OR "Allele Frequencies" OR "Frequencies, Allele" OR "Frequency, Allele" OR "Genetic Equilibrium" OR "Equilibrium, Genetic") OR ("allelic frequency") OR ("gene variant" OR "gene variants" OR "genetic variants" OR "genetic variant") OR ("Genetic Variation" OR "Genetic Variations" OR "Variations, Genetic" OR "Variation, Genetic" OR "Diversity, Genetic" OR "Diversities, Genetic" OR "Genetic Diversities" OR "Genetic Diversity") OR (pharmacogenetics OR pharmacogenomics)) | 04 |
| EBSCOHOST | TI ( Dipyrone OR Methamizole OR Metamizol OR Dipyronium OR Metamizole OR Biopyrin OR Novalgetol OR Novalgin OR Pyralgin OR Novaminsulfone OR Sulpyrin OR Sulpyrine OR Optalgin OR "Noramidopyrine Methanesulfonate Sodium" OR Novamidazophen OR "Metamizole Sodium" OR Methampyrone OR Algopyrin OR Analgin OR Narone OR "Noramidopyrine Methanesulfonate" ) OR AB ( Dipyrone OR Methamizole OR Metamizol OR Dipyronium OR Metamizole OR Biopyrin OR Novalgetol OR Novalgin OR Pyralgin OR Novaminsulfone OR Sulpyrin OR Sulpyrine OR Optalgin OR "Noramidopyrine Methanesulfonate Sodium" OR Novamidazophen OR "Metamizole Sodium" OR Methampyrone OR Algopyrin OR Analgin OR Narone OR "Noramidopyrine Methanesulfonate" ) AND TI ( Agranulocytosis OR Agranulocytoses OR Granulocytopenia OR Granulocytopenias ) OR AB ( Agranulocytosis OR Agranulocytoses OR Granulocytopenia OR Granulocytopenias ) OR TI ( Granulopenia OR panleukopenia ) OR AB ( Granulopenia OR panleukopenia ) OR TI ( Neutropenia OR Neutropenias ) OR AB ( Neutropenia OR Neutropenias ) OR TI ( "cyclic neutropaenia" OR "cyclic neutropenia" OR neutropaenia ) OR AB ( "cyclic neutropaenia" OR "cyclic neutropenia" OR neutropaenia ) OR TI ( Cytopenia OR Cytopenias ) OR AB (Cytopaenia) OR TI (Cytopaenia) AND TI ( "Polymorphism, Single Nucleotide" OR "Nucleotide Polymorphism, Single" OR "Nucleotide Polymorphisms, Single" OR "Polymorphisms, Single Nucleotide" OR "Single Nucleotide Polymorphisms" OR "SNPs" OR "Single Nucleotide Polymorphism" ) OR AB ( "Polymorphism, Single Nucleotide" OR "Nucleotide Polymorphism, Single" OR "Nucleotide Polymorphisms, Single" OR "Polymorphisms, Single Nucleotide" OR "Single Nucleotide Polymorphisms" OR "SNPs" OR "Single Nucleotide Polymorphism" ) OR TI ( "single nucleotide variant" OR "single nucleotide variation" ) OR AB ( "single nucleotide variant" OR "single nucleotide variation" ) OR TI ( "Polymorphism, Genetic" OR "Polymorphisms, Genetic" OR "Genetic Polymorphisms" OR "Genetic Polymorphism" OR "Polymorphism (Genetics)" OR "Polymorphisms (Genetics)" ) OR AB ( "Polymorphism, Genetic" OR "Polymorphisms, Genetic" OR "Genetic Polymorphisms" OR "Genetic Polymorphism" OR "Polymorphism (Genetics)" OR "Polymorphisms (Genetics)" ) OR TI ( Polymorphism OR Polymorphisms ) OR AB ( Polymorphism OR Polymorphisms ) OR TI ( "Gene Frequency" OR "Frequencies, Gene" OR "Frequency, Gene" OR "Gene Frequencies" OR "Allele Frequency" OR "Allele Frequencies" OR "Frequencies, Allele" OR "Frequency, Allele" OR "Genetic Equilibrium" OR "Equilibrium, Genetic" ) OR AB ( "Gene Frequency" OR "Frequencies, Gene" OR "Frequency, Gene" OR "Gene Frequencies" OR "Allele Frequency" OR "Allele Frequencies" OR "Frequencies, Allele" OR "Frequency, Allele" OR "Genetic Equilibrium" OR "Equilibrium, Genetic" ) OR TI "allelic frequency" OR AB "allelic frequency" OR TI ( "gene variant" OR "gene variants" OR "genetic variants" OR "genetic variant" ) OR AB ( "gene variant" OR "gene variants" OR "genetic variants" OR "genetic variant" ) OR TI ( "Genetic Variation" OR "Genetic Variations" OR "Variations, Genetic" OR "Variation, Genetic" OR "Diversity, Genetic" OR "Diversities, Genetic" OR "Genetic Diversities" OR "Genetic Diversity" ) OR AB ( "Genetic Variation" OR "Genetic Variations" OR "Variations, Genetic" OR "Variation, Genetic" OR "Diversity, Genetic" OR "Diversities, Genetic" OR "Genetic Diversities" OR "Genetic Diversity" ) OR TI ( Pharmacogenetics OR Pharmacogenomics ) OR AB ( Pharmacogenetics OR Pharmacogenomics ) | 04 |
| SCOPUS | ( TITLE-ABS-KEY ( dipyrone OR methamizole OR metamizol OR dipyronium OR metamizole OR biopyrin OR novalgetol OR novalgin OR pyralgin OR novaminsulfone OR sulpyrin OR sulpyrine OR optalgin OR "Noramidopyrine Methanesulfonate Sodium" OR novamidazophen OR "Metamizole Sodium" OR methampyrone OR algopyrin OR analgin OR narone OR "Noramidopyrine Methanesulfonate" ) ) AND ( ( TITLE-ABS-KEY ( agranulocytosis OR agranulocytoses OR granulocytopenia OR granulocytopenias ) OR TITLE-ABS-KEY ( granulopenia OR panleukopenia ) OR TITLE-ABS-KEY ( neutropenia OR neutropenias ) OR TITLE-ABS-KEY ( "cyclic neutropaenia" OR "cyclic neutropenia" OR neutropaenia ) OR TITLE-ABS-KEY ( cytopenia OR cytopenias ) OR TITLE-ABS-KEY ( cytopaenia ) ) ) AND ( ( TITLE-ABS-KEY ( "Polymorphism, Single Nucleotide" OR "Nucleotide Polymorphism, Single" OR "Nucleotide Polymorphisms, Single" OR "Polymorphisms, Single Nucleotide" OR "Single Nucleotide Polymorphisms" OR "SNPs" OR "Single Nucleotide Polymorphism" ) OR TITLE-ABS-KEY ( "single nucleotide variant" OR "single nucleotide variation" ) OR TITLE-ABS-KEY ( "Polymorphism, Genetic" OR "Polymorphisms, Genetic" OR "Genetic Polymorphisms" OR "Genetic Polymorphism" OR "Polymorphism (Genetics)" OR "Polymorphisms (Genetics)" ) OR TITLE-ABS-KEY ( polymorphism OR polymorphisms ) OR TITLE-ABS-KEY ( "Gene Frequency" OR "Frequencies, Gene" OR "Frequency, Gene" OR "Gene Frequencies" OR "Allele Frequency" OR "Allele Frequencies" OR "Frequencies, Allele" OR "Frequency, Allele" OR "Genetic Equilibrium" OR "Equilibrium, Genetic" ) OR TITLE-ABS-KEY ( "allelic frequency" ) OR TITLE-ABS-KEY ( "gene variant" OR "gene variants" OR "genetic variants" OR "genetic variant" ) OR TITLE-ABS-KEY ( "Genetic Variation" OR "Genetic Variations" OR "Variations, Genetic" OR "Variation, Genetic" OR "Diversity, Genetic" OR "Diversities, Genetic" OR "Genetic Diversities" OR "Genetic Diversity" ) OR TITLE-ABS-KEY ( pharmacogenetics OR pharmacogenomics ) ) ) | 16 |
| WEB OF SCIENCE | Dipyrone OR Methamizole OR Metamizol OR Dipyronium OR Metamizole OR Biopyrin OR Novalgetol OR Novalgin OR Pyralgin OR Novaminsulfone OR Sulpyrin OR Sulpyrine OR Optalgin OR "Noramidopyrine Methanesulfonate Sodium" OR Novamidazophen OR "Metamizole Sodium" OR Methampyrone OR Algopyrin OR Analgin OR Narone OR "Noramidopyrine Methanesulfonate" (Topic) and Preprint Citation Index (Exclude – Database) AND Agranulocytosis OR Agranulocytoses OR Granulocytopenia OR Granulocytopenias (Topic) or Granulopenia OR panleukopenia (Topic) or Neutropenia OR Neutropenias (Topic) or "cyclic neutropaenia" OR "cyclic neutropenia" OR neutropaenia (Topic) or Cytopenia OR Cytopenias (Topic) or Cytopaenia (Topic) and Preprint Citation Index (Exclude – Database) AND "Polymorphism, Single Nucleotide" OR "Nucleotide Polymorphism, Single" OR "Nucleotide Polymorphisms, Single" OR "Polymorphisms, Single Nucleotide" OR "Single Nucleotide Polymorphisms" OR "SNPs" OR "Single Nucleotide Polymorphism" (Topic) or "single nucleotide variant" OR "single nucleotide variation" (Topic) or "Polymorphism, Genetic" OR "Polymorphisms, Genetic" OR "Genetic Polymorphisms" OR "Genetic Polymorphism" OR "Polymorphism (Genetics)" OR "Polymorphisms (Genetics)" (Topic) or Polymorphism OR Polymorphisms (Topic) or "Gene Frequency" OR "Frequencies, Gene" OR "Frequency, Gene" OR "Gene Frequencies" OR "Allele Frequency" OR "Allele Frequencies" OR "Frequencies, Allele" OR "Frequency, Allele" OR "Genetic Equilibrium" OR "Equilibrium, Genetic" (Topic) or "allelic frequency" (Topic) or "gene variant" OR "gene variants" OR "genetic variants" OR "genetic variant" (Topic) or "Genetic Variation" OR "Genetic Variations" OR "Variations, Genetic" OR "Variation, Genetic" OR "Diversity, Genetic" OR "Diversities, Genetic" OR "Genetic Diversities" OR "Genetic Diversity" (Topic) or Pharmacogenetics OR Pharmacogenomics (Topic) and Preprint Citation Index (Exclude – Database) | 07 |
| EMBASE | 'dipyrone'/syn AND ('agranulocytosis'/syn OR 'neutropenia'/syn OR 'cytopenia'/syn) AND ('single nucleotide polymorphism'/syn OR 'genetic polymorphism'/syn OR polymorphism:ti,ab,kw OR polymorphisms:ti,ab,kw OR 'gene frequency'/syn OR 'gene variant':ti,ab,kw OR 'gene variants':ti,ab,kw OR 'genetic variants':ti,ab,kw OR 'genetic variant':ti,ab,kw OR 'genetic variation'/syn OR 'pharmacogenetics'/syn) | 22 |
| PROQUEST | (abstract(Dipyrone OR Methamizole OR Metamizol OR Dipyronium OR Metamizole OR Biopyrin OR Novalgetol OR Novalgin OR Pyralgin OR Novaminsulfone OR Sulpyrin OR Sulpyrine OR Optalgin OR "Noramidopyrine Methanesulfonate Sodium" OR Novamidazophen OR "Metamizole Sodium" OR Methampyrone OR Algopyrin OR Analgin OR Narone OR "Noramidopyrine Methanesulfonate") OR title(Dipyrone OR Methamizole OR Metamizol OR Dipyronium OR Metamizole OR Biopyrin OR Novalgetol OR Novalgin OR Pyralgin OR Novaminsulfone OR Sulpyrin OR Sulpyrine OR Optalgin OR "Noramidopyrine Methanesulfonate Sodium" OR Novamidazophen OR "Metamizole Sodium" OR Methampyrone OR Algopyrin OR Analgin OR Narone OR "Noramidopyrine Methanesulfonate")) AND (abstract(Agranulocytosis OR Agranulocytoses OR Granulocytopenia OR Granulocytopenias) OR title(Agranulocytosis OR Agranulocytoses OR Granulocytopenia OR Granulocytopenias) OR abstract(Granulopenia OR panleukopenia) OR title(Granulopenia OR panleukopenia) OR abstract(Neutropenia OR Neutropenias) OR title(Neutropenia OR Neutropenias) OR abstract("cyclic neutropaenia" OR "cyclic neutropenia" OR neutropaenia) OR title("cyclic neutropaenia" OR "cyclic neutropenia" OR neutropaenia) OR abstract(Cytopenia OR Cytopenias OR Cytopaenia) OR title(Cytopenia OR Cytopenias OR Cytopaenia)) AND ((abstract("Polymorphism, Single Nucleotide" OR "Nucleotide Polymorphism, Single" OR "Nucleotide Polymorphisms, Single" OR "Polymorphisms, Single Nucleotide" OR "Single Nucleotide Polymorphisms" OR "SNPs" OR "Single Nucleotide Polymorphism") OR title("Polymorphism, Single Nucleotide" OR "Nucleotide Polymorphism, Single" OR "Nucleotide Polymorphisms, Single" OR "Polymorphisms, Single Nucleotide" OR "Single Nucleotide Polymorphisms" OR "SNPs" OR "Single Nucleotide Polymorphism") OR abstract("single nucleotide variant" OR "single nucleotide variation") OR title("single nucleotide variant" OR "single nucleotide variation") OR abstract("Polymorphism, Genetic" OR "Polymorphisms, Genetic" OR "Genetic Polymorphisms" OR "Genetic Polymorphism" OR "Polymorphism (Genetics)" OR "Polymorphisms (Genetics)") OR title("Polymorphism, Genetic" OR "Polymorphisms, Genetic" OR "Genetic Polymorphisms" OR "Genetic Polymorphism" OR "Polymorphism (Genetics)" OR "Polymorphisms (Genetics)") OR abstract(Polymorphism OR Polymorphisms) OR title(Polymorphism OR Polymorphisms) OR abstract("Gene Frequency" OR "Frequencies, Gene" OR "Frequency, Gene" OR "Gene Frequencies" OR "Allele Frequency" OR "Allele Frequencies" OR "Frequencies, Allele" OR "Frequency, Allele" OR "Genetic Equilibrium" OR "Equilibrium, Genetic") OR title("Gene Frequency" OR "Frequencies, Gene" OR "Frequency, Gene" OR "Gene Frequencies" OR "Allele Frequency" OR "Allele Frequencies" OR "Frequencies, Allele" OR "Frequency, Allele" OR "Genetic Equilibrium" OR "Equilibrium, Genetic")) OR (abstract("allelic frequency") OR title("allelic frequency") OR abstract("gene variant" OR "gene variants" OR "genetic variants" OR "genetic variant") OR title("gene variant" OR "gene variants" OR "genetic variants" OR "genetic variant") OR abstract("Genetic Variation" OR "Genetic Variations" OR "Variations, Genetic" OR "Variation, Genetic" OR "Diversity, Genetic" OR "Diversities, Genetic" OR "Genetic Diversities" OR "Genetic Diversity") OR title("Genetic Variation" OR "Genetic Variations" OR "Variations, Genetic" OR "Variation, Genetic" OR "Diversity, Genetic" OR "Diversities, Genetic" OR "Genetic Diversities" OR "Genetic Diversity") OR abstract(Pharmacogenetics OR Pharmacogenomics) OR title(Pharmacogenetics OR Pharmacogenomics))) | 0 |
| Google Academic | Dipyrone AND (Agranulocytosis OR Neutropenia OR Cytopenia) AND ("Polymorphism, Single Nucleotide" OR "Nucleotide Polymorphism, Single" OR "Nucleotide Polymorphisms, Single" OR "Polymorphisms, Single Nucleotide" OR "Single Nucleotide Polymorphisms" OR "SNPs" OR "Single Nucleotide Polymorphism" OR "single nucleotide variant" OR "single nucleotide variation" OR "Polymorphism, Genetic" OR "Polymorphisms, Genetic" OR "Genetic Polymorphisms" OR "Genetic Polymorphism" OR "Polymorphism (Genetics)" OR "Polymorphisms (Genetics)" OR Polymorphism OR Polymorphisms OR "Gene Frequency" OR "Frequencies, Gene" OR "Frequency, Gene" OR "Gene Frequencies" OR "Allele Frequency" OR "Allele Frequencies" OR "Frequencies, Allele" OR "Frequency, Allele" OR "Genetic Equilibrium" OR "Equilibrium, Genetic" OR "allelic frequency" OR "gene variant" OR "gene variants" OR "genetic variants" OR "genetic variant" OR "Genetic Variation" OR "Genetic Variations" OR "Variations, Genetic" OR "Variation, Genetic" OR "Diversity, Genetic" OR "Diversities, Genetic" OR "Genetic Diversities" OR "Genetic Diversity" OR Pharmacogenetics OR Pharmacogenomics) | 123 |
| **Total** | | **182** |

**Table S3.** Excluded Studies after eligibility assessment

| Absolut Number | Reason for exclusion | Authors, year | Title | Reference |
| --- | --- | --- | --- | --- |
| 1 | Wrong outcome | Allgaier R, *et al*., 2023 | Case Report: Simultaneously Induced Neutropenia and Hemolysis After a Single Metamizole Dose | (Allgaier et al., 2023) |
| 2 | Wrong outcome | Zanrosso C, *et al.,* 2010 | N-Acetyltransferase 2 Polymorphisms and Susceptibility to Infant Leukemia with Maternal Exposure to Dipyrone during Pregnancy | (Zanrosso et al., 2010) |
| 3 | Wrong publication  type | Cismaru A, *et al.,* 2019 | Genetic case-control association study of metamizole-induced agranulocytosis | (Cismaru A.L., 2019) |

Allgaier, R., Kandulski, A., Gülow, K., Maier, L., Müller, M., and Tews, H. C. (2023). Case Report: Simultaneously Induced Neutropenia and Hemolysis After a Single Metamizole Dose. *Drugs R D* 23, 93–98. doi: 10.1007/s40268-023-00415-9

Cismaru A.L., R. D. , I. L. , L. E. , K. R. , C. A. , L. M. I. , M. J. , P. E. S. , E. N. , L. C. , H. M. , H. P. , W. M. , A. U. (2019). SGKC/SSCC Annual Assembly 2019 28th-30th August 2019 Technopark, Zurich or Evidence Based Laboratory Medicine. *Clinical Chemistry and Laboratory Medicine (CCLM)* 57, eA155–eA165. doi: 10.1515/cclm-2019-0735

Zanrosso, C. W., Emerenciano, M., Gonçalves, B. A. de A., Faro, A., Koifman, S., and Pombo-de-Oliveira, M. S. (2010). N-acetyltransferase 2 polymorphisms and susceptibility to infant leukemia with maternal exposure to dipyrone during pregnancy. *Cancer Epidemiology, Biomarkers & Prevention* 19, 3037–3043. doi: 10.1158/1055-9965.EPI-10-0508

**Table S4.** Legal status of metamizole in different countries.

| **Status** | **Country** |
| --- | --- |
| Over the counter | Argentina |
|  | Bolivia |
|  | Brazil |
|  | Cameroon |
|  | Chile |
|  | China |
|  | Egypt |
|  | Israel |
|  | Kazakhstan |
|  | Mexico |
|  | Mongolia |
|  | Paraguay |
|  | Poland |
|  | Russia |
|  | South Africa |
|  | Turkey |
|  | Uruguay |
| Per prescription | Belgium |
|  | Colombia |
|  | Germany |
|  | Italy |
|  | Peru |
|  | Portugal |
|  | Spain |
|  | Sudan |
|  | Switzerland |
|  | Thailand |
| Withdrawn | Åland Islands |
|  | Australia |
|  | Botswana |
|  | Canada |
|  | Denmark |
|  | England |
|  | Faroe Islands |
|  | Finland |
|  | France |
|  | Ghana |
|  | Greenland |
|  | Greenland |
|  | Iceland |
|  | India |
|  | Ireland |
|  | Japan |
|  | Kuwait |
|  | Malaysia |
|  | Morocco |
|  | Nigeria |
|  | Northern Ireland |
|  | Norway |
|  | Philippines |
|  | Saudi Arabia |
|  | Scotland |
|  | Svalbard |
|  | Sweden |
|  | United States |
|  | Venezuela |
|  | Wales |
|  | Zimbabwe |

Notes: Data from Withdrawn 2.0 platform (last update in October 2023) and review of the literature.

**Table S5.** rs55898176 allele frequency according to the genetic ancestry group.

| ***SVEP1* rs55898176** | | | | | |
| --- | --- | --- | --- | --- | --- |
| **Genetic Ancestry Group** | | **Allele Count** | **Allele Number** | **Number of Homozygotes** | **Allele Frequency** |
| Admixed American | *Overall* | 51 | 680 | 2 | 0.07500 |
|  | Colombians from Medellin, Colombia | 25 | 188 | 1 | 0.1330 |
|  | Mexican Ancestry from Los Angeles, USA | 6 | 124 | 1 | 0.04839 |
|  | Peruvians from Lima, Peru | 7 | 170 | 0 | 0.04118 |
|  | Puerto Ricans from Puerto Rico | 13 | 198 | 0 | 0.06566 |
| African | *Overall* | 6 | 1286 | 0 | 0.004666 |
|  | African Caribbeans in Barbados | 2 | 184 | 0 | 0.01087 |
|  | Americans of African Ancestry in SW USA | 2 | 102 | 0 | 0.01961 |
|  | Esan in Nigeria | 0 | 206 | 0 | 0.000 |
|  | Gambian in Western Divisions in the Gambia | 1 | 230 | 0 | 0.004348 |
|  | Luhya in Webuye, Kenya | 1 | 172 | 0 | 0.005814 |
|  | Mende in Sierra Leone | 0 | 160 | 0 | 0.000 |
|  | Yoruba in Ibadan, Nigeria | 0 | 232 | 0 | 0.000 |
| East Asian | *Overall* | 0 | 990 | 0 | 0.000 |
|  | Chinese Dai in Xishuangbanna, China | 0 | 176 | 0 | 0.000 |
|  | Han Chinese in Beijing, China | 0 | 204 | 0 | 0.000 |
|  | Japanese in Tokyo, Japan | 0 | 204 | 0 | 0.000 |
|  | Kinh in Ho Chi Minh City, Vietnam | 0 | 202 | 0 | 0.000 |
|  | Southern Han Chinese | 0 | 204 | 0 | 0.000 |
| European | *Overall* | 105 | 1022 | 7 | 0.1027 |
|  | British in England and Scotland | 17 | 174 | 1 | 0.09770 |
|  | Finnish in Finland | 17 | 196 | 0 | 0.08673 |
|  | Iberian Population in Spain | 23 | 208 | 2 | 0.1106 |
|  | Toscani in Italia | 27 | 206 | 3 | 0.1311 |
|  | Utah Residents (CEPH) with Northern and Western European Ancestry | 21 | 238 | 1 | 0.08824 |
| South Asian | *Overall* | 26 | 990 | 0 | 0.02626 |
|  | Bengali from Bangladesh | 3 | 198 | 0 | 0.01515 |
|  | Gujarati Indian from Houston, Texas | 9 | 200 | 0 | 0.04500 |
|  | Indian Telugu from the UK | 4 | 204 | 0 | 0.01961 |
|  | Punjabi from Lahore, Pakistan | 6 | 192 | 0 | 0.03125 |
|  | Sri Lankan Tamil from the UK | 4 | 196 | 0 | 0.02041 |
| **Total** |  | **188** | **4968** | **9** | **0.03784** |

Notes: Data from gnomAD browser, 1KG ([https://gnomad.broadinstitute.org](https://gnomad.broadinstitute.org/)), USA: United States of America, SW: southwest, CEPH: Centre d'Etude du Polymorphisme Humain, UK: United Kingdom.

**Table S6.** rs4427239 allele frequency according to the genetic ancestry group.

| **rs4427239** | | | | | |
| --- | --- | --- | --- | --- | --- |
| **Genetic Ancestry Group** | | **Allele Count** | **Allele Number** | **Number of Homozygotes** | **Allele Frequency** |
| Admixed American | *Overall* | 664 | 680 | 324 | 0.9765 |
|  | Colombians from Medellin, Colombia | 183 | 188 | 89 | 0.9734 |
|  | Mexican Ancestry from Los Angeles, USA | 122 | 124 | 60 | 0.9839 |
|  | Peruvians from Lima, Peru | 167 | 170 | 82 | 0.9824 |
|  | Puerto Ricans from Puerto Rico | 192 | 198 | 93 | 0.9697 |
| African | *Overall* | 1273 | 1286 | 630 | 0.9899 |
|  | African Caribbeans in Barbados | 183 | 184 | 91 | 0.9946 |
|  | Americans of African Ancestry in SW USA | 102 | 102 | 51 | 1.000 |
|  | Esan in Nigeria | 205 | 206 | 102 | 0.9951 |
|  | Gambian in Western Divisions in the Gambia | 224 | 230 | 109 | 0.9739 |
|  | Luhya in Webuye, Kenya | 171 | 172 | 85 | 0.9942 |
|  | Mende in Sierra Leone | 158 | 160 | 78 | 0.9875 |
|  | Yoruba in Ibadan, Nigeria | 230 | 232 | 114 | 0.9914 |
| East Asian | *Overall* | 691 | 990 | 244 | 0.6980 |
|  | Chinese Dai in Xishuangbanna, China | 109 | 176 | 34 | 0.6193 |
|  | Han Chinese in Beijing, China | 153 | 204 | 60 | 0.7500 |
|  | Japanese in Tokyo, Japan | 164 | 204 | 65 | 0.8039 |
|  | Kinh in Ho Chi Minh City, Vietnam | 140 | 202 | 49 | 0.6931 |
|  | Southern Han Chinese | 125 | 204 | 36 | 0.6127 |
| European | *Overall* | 983 | 1022 | 472 | 0.9618 |
|  | British in England and Scotland | 169 | 174 | 82 | 0.9713 |
|  | Finnish in Finland | 192 | 196 | 94 | 0.9796 |
|  | Iberian Population in Spain | 203 | 208 | 99 | 0.9760 |
|  | Toscani in Italia | 192 | 206 | 89 | 0.9320 |
|  | Utah Residents (CEPH) with Northern and Western European Ancestry | 227 | 238 | 108 | 0.9538 |
| South Asian | *Overall* | 828 | 990 | 349 | 0.8364 |
|  | Bengali from Bangladesh | 161 | 198 | 64 | 0.8131 |
|  | Gujarati Indian from Houston, Texas | 170 | 200 | 74 | 0.8500 |
|  | Indian Telugu from the UK | 169 | 204 | 70 | 0.8284 |
|  | Punjabi from Lahore, Pakistan | 169 | 192 | 76 | 0.8802 |
|  | Sri Lankan Tamil from the UK | 159 | 196 | 65 | 0.8112 |
| **Total** |  | **4439** | **4968** | **2019** | **0.8935** |

Notes: Data from gnomAD browser, 1KG ([https://gnomad.broadinstitute.org](https://gnomad.broadinstitute.org/)), USA: United States of America, SW: southwest, CEPH: Centre d'Etude du Polymorphisme Humain, UK: United Kingdom.

**Table S7.** *HLA-B*35:01* allele frequency.

| **Continent/Country** | **Sample size** | **Pooled number of altered alleles** | **Pooled number of alleles** | **Pooled allele frequency** |
| --- | --- | --- | --- | --- |
| **Africa** | **4167** | **411** | **8334** | **0.049** |
| Cameroon | 366 | 60 | 732 | 0.082 |
| Cape Verde | 124 | 33 | 248 | 0.132 |
| Ghana | 131 | 17 | 262 | 0.065 |
| Guinea | 65 | 19 | 130 | 0.144 |
| Ivory Coast | 44 | 5 | 88 | 0.057 |
| Kenya | 749 | 47 | 1498 | 0.031 |
| Libya | 118 | 9 | 236 | 0.038 |
| Mali | 138 | 35 | 276 | 0.127 |
| Morocco | 269 | 20 | 538 | 0.036 |
| Sao Tome and Principe | 98 | 11 | 196 | 0.056 |
| Senegal | 165 | 40 | 330 | 0.122 |
| South Africa | 654 | 42 | 1308 | 0.032 |
| Sudan | 200 | 17 | 400 | 0.043 |
| Tanzania | 336 | 24 | 672 | 0.036 |
| Tunisia | 100 | 10 | 200 | 0.048 |
| Uganda | 336 | 16 | 672 | 0.023 |
| Zambia | 44 | 2 | 88 | 0.023 |
| Zimbabwe | 230 | 6 | 460 | 0.013 |
| **Asia** | **374189** | **32615** | **748378** | **0.044** |
| China | 12879 | 661 | 25758 | 0.026 |
| Gaza | 42 | 3 | 84 | 0.036 |
| Hong Kong | 17322 | 630 | 34644 | 0.018 |
| India | 33881 | 3576 | 67762 | 0.053 |
| Indonesia | 237 | 2 | 474 | 0.005 |
| Iran | 437 | 136 | 874 | 0.155 |
| Israel | 195052 | 18989 | 390104 | 0.049 |
| Japan | 20160 | 3318 | 40320 | 0.082 |
| Jordan | 146 | 1 | 292 | 0.003 |
| Malaysia | 1544 | 91 | 3088 | 0.030 |
| Oman | 118 | 18 | 236 | 0.076 |
| Pakistan | 389 | 30 | 778 | 0.039 |
| Philippines | 50 | 0 | 100 | 0.000 |
| Russia | 5280 | 504 | 10560 | 0.048 |
| Saudi Arabia | 29298 | 1624 | 58596 | 0.028 |
| Singapore | 797 | 36 | 1594 | 0.023 |
| South Korea | 5098 | 452 | 10196 | 0.044 |
| Sri Lanka | 714 | 80 | 1428 | 0.056 |
| Taiwan | 49661 | 2410 | 99322 | 0.024 |
| Thailand | 191 | 8 | 382 | 0.021 |
| United Arab Emirates | 622 | 40 | 1244 | 0.033 |
| Vietnam | 271 | 5 | 542 | 0.009 |
| **Europe** | **3655017** | **425503** | **7310034** | **0.058** |
| Armenia | 100 | 19 | 200 | 0.095 |
| Austria | 200 | 27 | 400 | 0.068 |
| Azores | 232 | 31 | 464 | 0.067 |
| Bulgaria | 55 | 5 | 110 | 0.046 |
| Croatia | 4150 | 508 | 8300 | 0.061 |
| Czech Republic | 5205 | 554 | 10410 | 0.053 |
| England | 817 | 70 | 1634 | 0.043 |
| Finland | 91 | 21 | 182 | 0.117 |
| France | 42753 | 3934 | 85506 | 0.046 |
| Georgia | 220 | 37 | 440 | 0.085 |
| Germany | 3525931 | 412369 | 7051862 | 0.058 |
| Greece | 325 | 56 | 650 | 0.087 |
| Ireland | 1250 | 128 | 2500 | 0.051 |
| Italy | 1072 | 195 | 2144 | 0.091 |
| Kosovo | 124 | 10 | 248 | 0.040 |
| Madeira | 185 | 14 | 370 | 0.038 |
| Netherlands | 1369 | 181 | 2738 | 0.066 |
| Poland | 44448 | 4578 | 88896 | 0.051 |
| Portugal | 275 | 39 | 550 | 0.071 |
| Romania | 348 | 70 | 696 | 0.101 |
| Serbia | 102 | 18 | 204 | 0.088 |
| Spain | 4649 | 427 | 9298 | 0.046 |
| Sweden | 284 | 32 | 568 | 0.056 |
| Switzerland | 20832 | 2179 | 41664 | 0.052 |
| **North America** | **2916514** | **349132** | **5833028** | **0.060** |
| Canada | 59 | 23 | 118 | 0.191 |
| Costa Rica | 595 | 123 | 1190 | 0.103 |
| Cuba | 112 | 12 | 224 | 0.054 |
| Guatemala | 19 | 7 | 38 | 0.184 |
| Mexico | 2006 | 566 | 4012 | 0.141 |
| Nicaragua | 494 | 82 | 988 | 0.083 |
| Panama | 462 | 88 | 924 | 0.095 |
| USA | 2912767 | 348232 | 5825534 | 0.060 |
| **Oceania** | **428** | **13** | **856** | **0.015** |
| Australia | 428 | 13 | 856 | 0.015 |
| **South America** | **4031** | **400** | **8062** | **0.050** |
| Argentina | 289 | 12 | 578 | 0.021 |
| Bolivia/Chile | 20 | 2 | 40 | 0.050 |
| Bolivia/Peru | 21 | 2 | 42 | 0.048 |
| Brazil | 1734 | 190 | 3468 | 0.055 |
| Chile | 156 | 9 | 312 | 0.029 |
| Colombia | 1546 | 137 | 3092 | 0.044 |
| Peru | 210 | 46 | 420 | 0.110 |
| Venezuela | 55 | 1 | 110 | 0.011 |
| **Global** | **6954346** | **808074** | **13908692** | **0.058** |

Notes: Data from Allele Frequency Net Database (<http://www.allelefrequencies.net/>). USA: United States of America.

**Table S8.** *HLA-C*04:01* allele frequency.

| **Continent/Country** | **Sample size** | **Pooled number of altered alleles** | **Pooled number of alleles** | **Pooled allele frequency** |
| --- | --- | --- | --- | --- |
| **Africa** | **3708** | **968** | **7416** | **0.130** |
| Cameroon | 274 | 91 | 548 | 0.166 |
| Central African Republic | 36 | 0 | 72 | 0.000 |
| Ghana | 131 | 64 | 262 | 0.244 |
| Kenya | 749 | 187 | 1498 | 0.125 |
| Mali | 138 | 59 | 276 | 0.213 |
| Morocco | 415 | 71 | 830 | 0.085 |
| Senegal | 165 | 64 | 330 | 0.195 |
| South Africa | 554 | 115 | 1108 | 0.104 |
| Sudan | 200 | 54 | 400 | 0.135 |
| Tanzania | 336 | 57 | 672 | 0.084 |
| Tunisia | 100 | 23 | 200 | 0.116 |
| Uganda | 336 | 101 | 672 | 0.151 |
| Zambia | 44 | 13 | 88 | 0.144 |
| Zimbabwe | 230 | 68 | 460 | 0.148 |
| **Asia** | **121236** | **21658** | **242472** | **0.089** |
| China | 10339 | 1056 | 20678 | 0.051 |
| Gaza | 42 | 15 | 84 | 0.179 |
| Hong Kong | 12861 | 1024 | 25722 | 0.040 |
| India | 33769 | 8241 | 67538 | 0.122 |
| Iran | 284 | 116 | 568 | 0.205 |
| Israel | 247 | 75 | 494 | 0.152 |
| Japan | 20835 | 1830 | 41670 | 0.044 |
| Jordan | 146 | 29 | 292 | 0.100 |
| Lebanon | 97 | 47 | 194 | 0.243 |
| Malaysia | 1416 | 262 | 2832 | 0.093 |
| Pakistan | 389 | 73 | 778 | 0.094 |
| Philippines | 50 | 13 | 100 | 0.130 |
| Russia | 5557 | 961 | 11114 | 0.086 |
| Saudi Arabia | 29298 | 7095 | 58596 | 0.121 |
| Singapore | 554 | 86 | 1108 | 0.078 |
| South Korea | 1779 | 117 | 3558 | 0.033 |
| Sri Lanka | 714 | 177 | 1428 | 0.124 |
| Taiwan | 1824 | 211 | 3648 | 0.058 |
| Thailand | 142 | 17 | 284 | 0.060 |
| United Arab Emirates | 622 | 184 | 1244 | 0.148 |
| Vietnam | 271 | 28 | 542 | 0.052 |
| **Europe** | **3608971** | **831860** | **7217942** | **0.115** |
| Azores | 130 | 40 | 260 | 0.155 |
| Bulgaria | 13 | 2 | 26 | 0.091 |
| Czech Republic | 5205 | 1335 | 10410 | 0.128 |
| England | 817 | 126 | 1634 | 0.077 |
| Finland | 91 | 24 | 182 | 0.133 |
| France | 230 | 23 | 460 | 0.050 |
| Georgia | 140 | 62 | 280 | 0.221 |
| Germany | 3526105 | 811045 | 7052210 | 0.115 |
| Greece | 325 | 103 | 650 | 0.158 |
| Ireland | 1250 | 193 | 2500 | 0.077 |
| Italy | 1490 | 489 | 2980 | 0.164 |
| Kosovo | 124 | 40 | 248 | 0.161 |
| Netherlands | 1369 | 279 | 2738 | 0.102 |
| Poland | 44448 | 10865 | 88896 | 0.122 |
| Portugal | 692 | 218 | 1384 | 0.157 |
| Spain | 5026 | 1288 | 10052 | 0.128 |
| Switzerland | 20912 | 5605 | 41824 | 0.134 |
| United Kingdom | 604 | 122 | 1208 | 0.101 |
| **North America** | **2913799** | **781001** | **5827598** | **0.134** |
| Canada | 59 | 15 | 118 | 0.129 |
| Costa Rica | 595 | 223 | 1190 | 0.187 |
| Guatemala | 19 | 7 | 38 | 0.184 |
| Mexico | 1133 | 541 | 2266 | 0.239 |
| Nicaragua | 494 | 148 | 988 | 0.150 |
| Panama | 462 | 180 | 924 | 0.195 |
| USA | 2911037 | 779887 | 5822074 | 0.134 |
| **Oceania** | **1373** | **410** | **2746** | **0.149** |
| American Samoa | 51 | 4 | 102 | 0.040 |
| Australia | 544 | 237 | 1088 | 0.218 |
| New Caledonia | 65 | 22 | 130 | 0.167 |
| New Zealand | 199 | 26 | 398 | 0.065 |
| Papua New Guinea | 514 | 121 | 1028 | 0.118 |
| **South America** | **3680** | **1020** | **7360** | **0.139** |
| Bolivia/Chile | 20 | 21 | 40 | 0.525 |
| Bolivia/Peru | 21 | 12 | 42 | 0.286 |
| Brazil | 1699 | 475 | 3398 | 0.140 |
| Chile | 111 | 12 | 222 | 0.053 |
| Colombia | 1642 | 468 | 3284 | 0.142 |
| Colombia/Brazil | 36 | 13 | 72 | 0.175 |
| Paraguay/Argentina | 23 | 6 | 46 | 0.130 |
| Venezuela | 128 | 14 | 256 | 0.054 |
| **Global** | **6652767** | **1636917** | **13305534** | **0.123** |

Notes: Data from Allele Frequency Net Database (<http://www.allelefrequencies.net/>). USA: United States of America.

**Table S9.** *HLA-C*07:04* allele frequency.

| **Continent/Country** | **Sample size** | **Pooled number of altered alleles** | **Pooled number of alleles** | **Pooled allele frequency** |
| --- | --- | --- | --- | --- |
| **Africa** | **3617** | **174** | **7234** | **0.024** |
| Burkina | 149 | 1 | 298 | 0.003 |
| Cameroon | 274 | 1 | 548 | 0.002 |
| Kenya | 749 | 68 | 1498 | 0.045 |
| Mali | 138 | 0 | 276 | 0.000 |
| Morocco | 342 | 0 | 684 | 0.000 |
| Senegal | 165 | 0 | 330 | 0.000 |
| South Africa | 554 | 24 | 1108 | 0.022 |
| Sudan | 200 | 6 | 400 | 0.015 |
| Tanzania | 336 | 39 | 672 | 0.058 |
| Tunisia | 100 | 2 | 200 | 0.010 |
| Uganda | 336 | 12 | 672 | 0.017 |
| Zambia | 44 | 2 | 88 | 0.022 |
| Zimbabwe | 230 | 19 | 460 | 0.042 |
| **Asia** | **116810** | **3056** | **233620** | **0.013** |
| China | 9620 | 155 | 19240 | 0.008 |
| Gaza | 42 | 1 | 84 | 0.012 |
| Hong Kong | 12861 | 147 | 25722 | 0.006 |
| India | 33566 | 1307 | 67132 | 0.019 |
| Japan | 20835 | 396 | 41670 | 0.010 |
| Lebanon | 97 | 1 | 194 | 0.005 |
| Malaysia | 1416 | 191 | 2832 | 0.067 |
| Pakistan | 1012 | 4 | 2024 | 0.002 |
| Philippines | 50 | 7 | 100 | 0.070 |
| Russia | 3923 | 145 | 7846 | 0.018 |
| Saudi Arabia | 29140 | 484 | 58280 | 0.008 |
| Singapore | 554 | 66 | 1108 | 0.060 |
| South Korea | 485 | 5 | 970 | 0.005 |
| Sri Lanka | 714 | 36 | 1428 | 0.025 |
| Taiwan | 970 | 16 | 1940 | 0.008 |
| Thailand | 542 | 63 | 1084 | 0.058 |
| Turkey | 142 | 7 | 284 | 0.025 |
| United Arab Emirates | 570 | 14 | 1140 | 0.012 |
| Vietnam | 271 | 10 | 542 | 0.019 |
| **Europe** | **3608454** | **147569** | **7216908** | **0.020** |
| Czech Republic | 5205 | 205 | 10410 | 0.020 |
| England | 1317 | 24 | 2634 | 0.009 |
| Finland | 91 | 1 | 182 | 0.006 |
| France | 230 | 6 | 460 | 0.013 |
| Georgia | 140 | 3 | 280 | 0.011 |
| Germany | 3526105 | 144185 | 7052210 | 0.020 |
| Greece | 325 | 13 | 650 | 0.020 |
| Ireland | 1250 | 33 | 2500 | 0.013 |
| Italy | 1393 | 34 | 2786 | 0.012 |
| Kosovo | 124 | 1 | 248 | 0.004 |
| Netherlands | 1369 | 35 | 2738 | 0.013 |
| Poland | 44448 | 2191 | 88896 | 0.025 |
| Portugal | 130 | 5 | 260 | 0.018 |
| Scotland | 99 | 0 | 198 | 0.000 |
| Spain | 4712 | 75 | 9424 | 0.008 |
| Switzerland | 20912 | 735 | 41824 | 0.018 |
| United Kingdom | 604 | 24 | 1208 | 0.020 |
| **North America** | **2911308** | **67925** | **5822616** | **0.012** |
| Costa Rica | 456 | 8 | 912 | 0.009 |
| Mexico | 377 | 3 | 754 | 0.004 |
| Nicaragua | 494 | 5 | 988 | 0.005 |
| USA | 2909981 | 67909 | 5819962 | 0.012 |
| **Oceania** | **1116** | **14** | **2232** | **0.006** |
| Australia | 503 | 9 | 1006 | 0.009 |
| New Zealand | 613 | 5 | 1226 | 0.004 |
| **South America** | **2879** | **51** | **5758** | **0.009** |
| Brazil | 1416 | 38 | 2832 | 0.013 |
| Colombia | 1463 | 13 | 2926 | 0.004 |
| **Global** | **6644184** | **218788** | **13288368** | **0.016** |

Notes: Data from Allele Frequency Net Database (<http://www.allelefrequencies.net/>). USA: United States of America.

**Table S10.** *HLA-DQA1*01:02* allele frequency.

| **Continent/Country** | **Sample size** | **Pooled number of altered alleles** | **Pooled number of alleles** | **Pooled allele frequency** |
| --- | --- | --- | --- | --- |
| **Africa** | **2056** | **1237** | **4112** | **0.301** |
| Cameroon | 298 | 221 | 596 | 0.370 |
| Congo | 90 | 75 | 180 | 0.417 |
| Equatorial Guinea | 246 | 102 | 492 | 0.206 |
| Ethiopia | 181 | 103 | 362 | 0.285 |
| Gabon | 167 | 167 | 334 | 0.500 |
| Kenya | 244 | 151 | 488 | 0.310 |
| Morocco | 294 | 121 | 588 | 0.205 |
| South Africa | 159 | 79 | 318 | 0.248 |
| Tunisia | 100 | 24 | 200 | 0.121 |
| Uganda | 47 | 37 | 94 | 0.394 |
| Zimbabwe | 230 | 158 | 460 | 0.343 |
| **Asia** | **23502** | **6587** | **47004** | **0.140** |
| China | 2115 | 658 | 4230 | 0.155 |
| Hong Kong | 6330 | 1681 | 12660 | 0.133 |
| India | 1792 | 406 | 3584 | 0.113 |
| Indonesia | 62 | 16 | 124 | 0.130 |
| Iran | 58 | 14 | 116 | 0.121 |
| Israel | 932 | 236 | 1864 | 0.127 |
| Japan | 4660 | 1160 | 9320 | 0.124 |
| Jordan | 146 | 34 | 292 | 0.117 |
| Mongolia | 126 | 14 | 252 | 0.054 |
| Pakistan | 389 | 86 | 778 | 0.111 |
| Russia | 3302 | 829 | 6604 | 0.126 |
| Singapore | 271 | 274 | 542 | 0.505 |
| South Korea | 1209 | 353 | 2418 | 0.146 |
| Sri Lanka | 714 | 188 | 1428 | 0.132 |
| Taiwan | 65 | 14 | 130 | 0.108 |
| Thailand | 459 | 198 | 918 | 0.216 |
| Turkey | 250 | 82 | 500 | 0.164 |
| United Arab Emirates | 622 | 344 | 1244 | 0.276 |
| **Europe** | **8370** | **2369** | **16740** | **0.142** |
| Azores | 130 | 28 | 260 | 0.109 |
| Croatia | 354 | 130 | 708 | 0.184 |
| Czech Republic | 419 | 109 | 838 | 0.130 |
| Denmark | 55 | 24 | 110 | 0.218 |
| England | 889 | 125 | 1778 | 0.070 |
| France | 569 | 243 | 1138 | 0.214 |
| Georgia | 80 | 21 | 160 | 0.131 |
| Germany | 285 | 79 | 570 | 0.139 |
| Greece | 1012 | 370 | 2024 | 0.183 |
| Italy | 910 | 260 | 1820 | 0.143 |
| Kosovo | 124 | 66 | 248 | 0.266 |
| Macedonia | 103 | 61 | 206 | 0.296 |
| Netherlands | 155 | 0 | 310 | 0.000 |
| Norway | 181 | 80 | 362 | 0.220 |
| Poland | 202 | 77 | 404 | 0.191 |
| Portugal | 692 | 172 | 1384 | 0.125 |
| Scotland | 99 | 45 | 198 | 0.225 |
| Slovenia | 240 | 0 | 480 | 0.000 |
| Spain | 1488 | 354 | 2976 | 0.119 |
| Sweden | 383 | 124 | 766 | 0.162 |
| **North America** | **6444** | **945** | **12888** | **0.073** |
| Canada | 62 | 0 | 124 | 0.000 |
| Jamaica | 132 | 55 | 264 | 0.208 |
| Mexico | 849 | 78 | 1698 | 0.046 |
| Nicaragua | 339 | 90 | 678 | 0.133 |
| USA | 5062 | 722 | 10124 | 0.071 |
| **Oceania** | **804** | **314** | **1608** | **0.195** |
| Australia | 144 | 32 | 288 | 0.110 |
| Cook Islands | 78 | 7 | 156 | 0.045 |
| Fiji | 57 | 25 | 114 | 0.219 |
| Kiribati | 62 | 16 | 124 | 0.129 |
| Nauru | 67 | 44 | 134 | 0.328 |
| New Caledonia | 65 | 32 | 130 | 0.246 |
| Niue | 70 | 5 | 140 | 0.036 |
| Papua New Guinea | 210 | 143 | 420 | 0.341 |
| Western Samoa/Tokelau | 51 | 10 | 102 | 0.098 |
| **South America** | **3367** | **615** | **6734** | **0.091** |
| Argentina | 809 | 164 | 1618 | 0.101 |
| Brazil | 1812 | 308 | 3624 | 0.085 |
| Colombia | 295 | 74 | 590 | 0.126 |
| Ecuador | 320 | 65 | 640 | 0.102 |
| Paraguay | 87 | 0 | 174 | 0.000 |
| Peru | 44 | 4 | 88 | 0.045 |
| **Global** | **44543** | **12067** | **89086** | **0.135** |

Notes: Data from Allele Frequency Net Database (<http://www.allelefrequencies.net/>). USA: United States of America.

**Table S11.** *HLA-DQB1*05:01* allele frequency.

| **Continent/Country** | **Sample size** | **Pooled number of altered alleles** | **Pooled number of alleles** | **Pooled allele frequency** |
| --- | --- | --- | --- | --- |
| **Africa** | **5594** | **1598** | **11188** | **0.143** |
| Algeria | 206 | 14 | 412 | 0.034 |
| Cameroon | 298 | 69 | 596 | 0.116 |
| Central African Republic | 93 | 39 | 186 | 0.208 |
| Congo | 90 | 35 | 180 | 0.194 |
| Equatorial Guinea | 100 | 42 | 200 | 0.210 |
| Ethiopia | 181 | 45 | 362 | 0.124 |
| Gabon | 167 | 49 | 334 | 0.147 |
| Gambia | 1085 | 462 | 2170 | 0.213 |
| Kenya | 100 | 34 | 200 | 0.170 |
| Morocco | 390 | 93 | 780 | 0.119 |
| Rwanda | 561 | 219 | 1122 | 0.195 |
| South Africa | 786 | 66 | 1572 | 0.042 |
| Sudan | 200 | 57 | 400 | 0.143 |
| Tanzania | 336 | 95 | 672 | 0.142 |
| Tunisia | 724 | 150 | 1448 | 0.104 |
| Uganda | 47 | 24 | 94 | 0.255 |
| Zimbabwe | 230 | 104 | 460 | 0.227 |
| **Asia** | **105623** | **18565** | **211246** | **0.088** |
| China | 9933 | 849 | 19866 | 0.043 |
| Gaza | 165 | 39 | 330 | 0.118 |
| Hong Kong | 6474 | 358 | 12948 | 0.028 |
| India | 35230 | 6638 | 70460 | 0.094 |
| Indonesia | 135 | 47 | 270 | 0.175 |
| Iran | 953 | 206 | 1906 | 0.108 |
| Israel | 932 | 189 | 1864 | 0.102 |
| Japan | 4723 | 618 | 9446 | 0.065 |
| Jordan | 146 | 27 | 292 | 0.093 |
| Lebanon | 428 | 140 | 856 | 0.164 |
| Malaysia | 1565 | 368 | 3130 | 0.118 |
| Mongolia | 574 | 92 | 1148 | 0.081 |
| Pakistan | 389 | 54 | 778 | 0.069 |
| Russia | 7003 | 1614 | 14006 | 0.115 |
| Saudi Arabia | 29366 | 5768 | 58732 | 0.098 |
| Singapore | 403 | 112 | 806 | 0.139 |
| South Korea | 1227 | 189 | 2454 | 0.077 |
| Sri Lanka | 714 | 146 | 1428 | 0.102 |
| Taiwan | 933 | 63 | 1866 | 0.034 |
| Thailand | 925 | 260 | 1850 | 0.140 |
| Turkey | 250 | 41 | 500 | 0.082 |
| United Arab Emirates | 622 | 110 | 1244 | 0.088 |
| Vietnam | 2533 | 637 | 5066 | 0.126 |
| **Europe** | **3535821** | **823990** | **7071642** | **0.117** |
| Austria | 200 | 55 | 400 | 0.138 |
| Azores | 130 | 29 | 260 | 0.113 |
| Belgium | 814 | 287 | 1628 | 0.176 |
| Cretan Islanders | 124 | 34 | 248 | 0.137 |
| Croatia | 354 | 119 | 708 | 0.168 |
| Czech Republic | 5518 | 1116 | 11036 | 0.101 |
| Denmark | 55 | 15 | 110 | 0.138 |
| England | 536 | 128 | 1072 | 0.120 |
| Finland | 91 | 21 | 182 | 0.114 |
| France | 1104 | 215 | 2208 | 0.098 |
| Georgia | 199 | 53 | 398 | 0.133 |
| Germany | 3462366 | 810533 | 6924732 | 0.117 |
| Greece | 1472 | 241 | 2944 | 0.082 |
| Ireland | 372 | 65 | 744 | 0.087 |
| Italy | 7589 | 486 | 15178 | 0.032 |
| Kosovo | 124 | 14 | 248 | 0.057 |
| Macedonia | 355 | 62 | 710 | 0.087 |
| Madeira | 173 | 59 | 346 | 0.170 |
| Netherlands | 1816 | 371 | 3632 | 0.102 |
| Norway | 181 | 36 | 362 | 0.100 |
| Poland | 23997 | 4097 | 47994 | 0.085 |
| Portugal | 692 | 176 | 1384 | 0.127 |
| Slovakia | 146 | 33 | 292 | 0.112 |
| Slovenia | 240 | 0 | 480 | 0.000 |
| Spain | 5958 | 1458 | 11916 | 0.122 |
| Sweden | 383 | 99 | 766 | 0.129 |
| Switzerland | 20832 | 4187 | 41664 | 0.100 |
| **North America** | **239857** | **53783** | **479714** | **0.112** |
| Canada | 62 | 1 | 124 | 0.008 |
| Costa Rica | 221 | 70 | 442 | 0.158 |
| Guatemala | 132 | 8 | 264 | 0.030 |
| Martinique | 100 | 32 | 200 | 0.160 |
| Mexico | 2749 | 440 | 5498 | 0.080 |
| Nicaragua | 339 | 69 | 678 | 0.102 |
| Panama | 462 | 72 | 924 | 0.078 |
| USA | 235792 | 53091 | 471584 | 0.113 |
| **Oceania** | **1412** | **32** | **2824** | **0.011** |
| Australia | 103 | 6 | 206 | 0.030 |
| Cook Islands | 128 | 2 | 256 | 0.008 |
| Fiji | 57 | 2 | 114 | 0.018 |
| Kiribati | 62 | 2 | 124 | 0.016 |
| Nauru | 67 | 1 | 134 | 0.008 |
| New Caledonia | 65 | 2 | 130 | 0.015 |
| New Zealand | 200 | 14 | 400 | 0.034 |
| Niue | 70 | 0 | 140 | 0.000 |
| Papua New Guinea | 430 | 2 | 860 | 0.002 |
| Samoa | 79 | 0 | 158 | 0.000 |
| Tokelau | 50 | 0 | 100 | 0.000 |
| Tonga | 50 | 0 | 100 | 0.000 |
| Western Samoa and Tokelau | 51 | 1 | 102 | 0.010 |
| **South America** | **7184** | **1296** | **14368** | **0.090** |
| Argentina | 918 | 129 | 1836 | 0.070 |
| Bolivia | 156 | 2 | 312 | 0.007 |
| Brazil | 2999 | 672 | 5998 | 0.112 |
| Chile | 260 | 41 | 520 | 0.079 |
| Colombia | 2081 | 415 | 4162 | 0.100 |
| Ecuador | 178 | 24 | 356 | 0.067 |
| Paraguay | 87 | 0 | 174 | 0.000 |
| Peru | 505 | 14 | 1010 | 0.014 |
| **Global** | **3895491** | **899264** | **7790982** | **0.115** |

Notes: Data from Allele Frequency Net Database (<http://www.allelefrequencies.net/>). USA: United States of America.

**Table S12.** *HLA-DQB1*06:04* allele frequency.

| **Continent/Country** | **Sample size** | **Pooled number of altered alleles** | **Pooled number of alleles** | **Pooled allele frequency** |
| --- | --- | --- | --- | --- |
| **Africa** | **4842** | **422** | **9684** | **0.044** |
| Algeria | 206 | 11 | 412 | 0.027 |
| Cameroon | 298 | 9 | 596 | 0.015 |
| Central African Republic | 93 | 12 | 186 | 0.065 |
| Congo | 90 | 4 | 180 | 0.022 |
| Ethiopia | 181 | 38 | 362 | 0.105 |
| Gabon | 167 | 30 | 334 | 0.090 |
| Gambia | 1085 | 27 | 2170 | 0.012 |
| Kenya | 244 | 20 | 488 | 0.041 |
| Morocco | 390 | 25 | 780 | 0.032 |
| Rwanda | 561 | 120 | 1122 | 0.107 |
| South Africa | 301 | 17 | 602 | 0.028 |
| Tanzania | 336 | 65 | 672 | 0.097 |
| Tunisia | 613 | 28 | 1226 | 0.023 |
| Uganda | 47 | 5 | 94 | 0.053 |
| Zimbabwe | 230 | 11 | 460 | 0.024 |
| **Asia** | **101018** | **4910** | **202036** | **0.024** |
| China | 9175 | 211 | 18350 | 0.012 |
| Gaza | 165 | 13 | 330 | 0.039 |
| Hong Kong | 6330 | 42 | 12660 | 0.003 |
| India | 35139 | 963 | 70278 | 0.014 |
| Indonesia | 62 | 0 | 124 | 0.000 |
| Iran | 953 | 54 | 1906 | 0.028 |
| Israel | 772 | 60 | 1544 | 0.039 |
| Japan | 4723 | 524 | 9446 | 0.055 |
| Jordan | 146 | 8 | 292 | 0.028 |
| Lebanon | 237 | 13 | 474 | 0.027 |
| Malaysia | 1247 | 13 | 2494 | 0.005 |
| Mongolia | 574 | 20 | 1148 | 0.017 |
| Pakistan | 389 | 1 | 778 | 0.001 |
| Russia | 4489 | 171 | 8978 | 0.019 |
| Saudi Arabia | 29366 | 2621 | 58732 | 0.045 |
| South Korea | 1712 | 117 | 3424 | 0.034 |
| Sri Lanka | 714 | 11 | 1428 | 0.008 |
| Taiwan | 933 | 5 | 1866 | 0.003 |
| Thailand | 590 | 10 | 1180 | 0.009 |
| Turkey | 250 | 16 | 500 | 0.032 |
| United Arab Emirates | 622 | 26 | 1244 | 0.021 |
| Vietnam | 2430 | 11 | 4860 | 0.002 |
| **Europe** | **3530449** | **258710** | **7060898** | **0.037** |
| Austria | 200 | 22 | 400 | 0.055 |
| Azores | 260 | 6 | 520 | 0.012 |
| Cretan Islanders | 124 | 8 | 248 | 0.032 |
| Croatia | 303 | 15 | 606 | 0.025 |
| Czech Republic | 5518 | 272 | 11036 | 0.025 |
| Denmark | 55 | 5 | 110 | 0.046 |
| England | 536 | 27 | 1072 | 0.025 |
| Finland | 91 | 3 | 182 | 0.014 |
| France | 1104 | 53 | 2208 | 0.024 |
| Georgia | 199 | 15 | 398 | 0.038 |
| Germany | 3462366 | 254768 | 6924732 | 0.037 |
| Greece | 1472 | 73 | 2944 | 0.025 |
| Ireland | 772 | 28 | 1544 | 0.018 |
| Italy | 3014 | 140 | 6028 | 0.023 |
| Kosovo | 124 | 10 | 248 | 0.040 |
| Macedonia | 355 | 13 | 710 | 0.018 |
| Madeira | 173 | 12 | 346 | 0.035 |
| Netherlands | 1816 | 119 | 3632 | 0.033 |
| Norway | 181 | 18 | 362 | 0.050 |
| Poland | 23997 | 1109 | 47994 | 0.023 |
| Portugal | 692 | 32 | 1384 | 0.023 |
| Slovakia | 146 | 5 | 292 | 0.018 |
| Slovenia | 240 | 0 | 480 | 0.000 |
| Spain | 5496 | 285 | 10992 | 0.026 |
| Sweden | 383 | 33 | 766 | 0.043 |
| Switzerland | 20832 | 1641 | 41664 | 0.039 |
| **North America** | **243369** | **11253** | **486738** | **0.023** |
| Canada | 62 | 0 | 124 | 0.000 |
| Costa Rica | 221 | 13 | 442 | 0.030 |
| Guatemala | 132 | 2 | 264 | 0.007 |
| Jamaica | 132 | 18 | 264 | 0.068 |
| Martinique | 100 | 10 | 200 | 0.050 |
| Mexico | 2072 | 53 | 4144 | 0.013 |
| Nicaragua | 339 | 9 | 678 | 0.014 |
| Panama | 462 | 12 | 924 | 0.013 |
| USA | 239849 | 11136 | 479698 | 0.023 |
| **Oceania** | **556** | **14** | **1112** | **0.013** |
| Cook Islands | 78 | 2 | 156 | 0.013 |
| Kiribati | 62 | 0 | 124 | 0.000 |
| Nauru | 67 | 2 | 134 | 0.015 |
| New Zealand | 200 | 8 | 400 | 0.021 |
| Niue | 70 | 0 | 140 | 0.000 |
| Papua New Guinea | 28 | 0 | 56 | 0.000 |
| Western Samoa and Tokelau | 51 | 2 | 102 | 0.020 |
| **South America** | **6450** | **324** | **12900** | **0.025** |
| Argentina | 870 | 48 | 1740 | 0.027 |
| Bolivia | 156 | 3 | 312 | 0.010 |
| Brazil | 2939 | 173 | 5878 | 0.029 |
| Chile | 136 | 1 | 272 | 0.004 |
| Colombia | 1755 | 90 | 3510 | 0.026 |
| Ecuador | 133 | 3 | 266 | 0.011 |
| Peru | 461 | 6 | 922 | 0.007 |
| **Global** | **3886684** | **275634** | **7773368** | **0.035** |

Notes: Data from Allele Frequency Net Database (<http://www.allelefrequencies.net/>). USA: United States of America.

**Table S13.** *HLA-DRB1*13:02* allele frequency.

| **Continent/Country** | **Sample size** | **Pooled number of altered alleles** | **Pooled number of alleles** | **Pooled allele frequency** |
| --- | --- | --- | --- | --- |
| **Africa** | **6016** | **1000** | **12032** | **0.083** |
| Algeria | 303 | 17 | 606 | 0.028 |
| Cameroon | 126 | 11 | 252 | 0.043 |
| Cape Verde | 124 | 21 | 248 | 0.085 |
| Central African Republic | 93 | 10 | 186 | 0.054 |
| Congo | 165 | 41 | 330 | 0.124 |
| Equatorial Guinea | 100 | 13 | 200 | 0.063 |
| Ethiopia | 181 | 62 | 362 | 0.171 |
| Gambia | 939 | 270 | 1878 | 0.144 |
| Guinea | 65 | 10 | 130 | 0.077 |
| Kenya | 100 | 9 | 200 | 0.045 |
| Libya | 118 | 22 | 236 | 0.093 |
| Morocco | 489 | 73 | 978 | 0.075 |
| Nigeria | 258 | 36 | 516 | 0.070 |
| Rwanda | 561 | 158 | 1122 | 0.141 |
| Sao Tome and Principe | 98 | 3 | 196 | 0.015 |
| Senegal | 112 | 10 | 224 | 0.044 |
| South Africa | 1005 | 41 | 2010 | 0.020 |
| Tanzania | 336 | 119 | 672 | 0.177 |
| Tunisia | 613 | 44 | 1226 | 0.036 |
| Zimbabwe | 230 | 30 | 460 | 0.066 |
| **Asia** | **387355** | **39318** | **774710** | **0.051** |
| China | 15200 | 1058 | 30400 | 0.035 |
| Gaza | 207 | 19 | 414 | 0.046 |
| Hong Kong | 16753 | 621 | 33506 | 0.019 |
| India | 35367 | 1904 | 70734 | 0.027 |
| Indonesia | 658 | 8 | 1316 | 0.006 |
| Iran | 788 | 35 | 1576 | 0.022 |
| Israel | 195737 | 26906 | 391474 | 0.069 |
| Japan | 25448 | 2953 | 50896 | 0.058 |
| Jordan | 146 | 1 | 292 | 0.003 |
| Lebanon | 237 | 12 | 474 | 0.026 |
| Malaysia | 1717 | 70 | 3434 | 0.020 |
| Mongolia | 861 | 55 | 1722 | 0.032 |
| Pakistan | 912 | 22 | 1824 | 0.012 |
| Philippines | 84 | 0 | 168 | 0.000 |
| Russia | 4436 | 246 | 8872 | 0.028 |
| Saudi Arabia | 29416 | 3953 | 58832 | 0.067 |
| Singapore | 132 | 5 | 264 | 0.019 |
| South Korea | 6304 | 1144 | 12608 | 0.091 |
| Sri Lanka | 714 | 44 | 1428 | 0.031 |
| Taiwan | 49968 | 165 | 99936 | 0.002 |
| Thailand | 941 | 18 | 1882 | 0.009 |
| Turkey | 250 | 25 | 500 | 0.050 |
| United Arab Emirates | 622 | 39 | 1244 | 0.032 |
| Vietnam | 457 | 14 | 914 | 0.016 |
| **Europe** | **3665416** | **308558** | **7330832** | **0.042** |
| Armenia | 100 | 3 | 200 | 0.015 |
| Austria | 200 | 25 | 400 | 0.062 |
| Azores | 232 | 11 | 464 | 0.023 |
| Belgium | 99 | 7 | 198 | 0.035 |
| Bulgaria | 55 | 3 | 110 | 0.027 |
| Cretan Islanders | 124 | 10 | 248 | 0.040 |
| Croatia | 4263 | 375 | 8526 | 0.044 |
| Czech Republic | 5338 | 312 | 10676 | 0.029 |
| Denmark | 55 | 6 | 110 | 0.056 |
| England | 829 | 32 | 1658 | 0.019 |
| Finland | 241 | 14 | 482 | 0.029 |
| France | 43253 | 3906 | 86506 | 0.045 |
| Georgia | 199 | 17 | 398 | 0.043 |
| Germany | 3526216 | 298236 | 7052432 | 0.042 |
| Greece | 924 | 46 | 1848 | 0.025 |
| Ireland | 1250 | 78 | 2500 | 0.031 |
| Italy | 6281 | 143 | 12562 | 0.011 |
| Kosovo | 124 | 16 | 248 | 0.065 |
| Macedonia | 410 | 25 | 820 | 0.031 |
| Madeira | 358 | 30 | 716 | 0.042 |
| Netherlands | 1816 | 135 | 3632 | 0.037 |
| Norway | 181 | 18 | 362 | 0.050 |
| Poland | 44448 | 2282 | 88896 | 0.026 |
| Portugal | 405 | 21 | 810 | 0.026 |
| Scotland | 99 | 9 | 198 | 0.043 |
| Slovenia | 240 | 0 | 480 | 0.000 |
| Spain | 6560 | 510 | 13120 | 0.039 |
| Sweden | 284 | 21 | 568 | 0.037 |
| Switzerland | 20832 | 2267 | 41664 | 0.054 |
| **North America** | **2981617** | **287444** | **5963234** | **0.048** |
| Canada | 88 | 1 | 176 | 0.006 |
| Costa Rica | 577 | 46 | 1154 | 0.040 |
| Cuba | 78 | 10 | 156 | 0.061 |
| Guatemala | 151 | 5 | 302 | 0.016 |
| Martinique | 100 | 10 | 200 | 0.050 |
| Mexico | 2316 | 64 | 4632 | 0.014 |
| Nicaragua | 494 | 39 | 988 | 0.039 |
| Panama | 462 | 34 | 924 | 0.036 |
| USA | 2977351 | 287237 | 5954702 | 0.048 |
| **Oceania** | **909** | **15** | **1818** | **0.008** |
| Cook Islands | 78 | 3 | 156 | 0.019 |
| Kiribati | 62 | 0 | 124 | 0.000 |
| Nauru | 67 | 2 | 134 | 0.015 |
| New Zealand | 332 | 8 | 664 | 0.013 |
| Niue | 70 | 0 | 140 | 0.000 |
| Papua New Guinea | 220 | 0 | 440 | 0.000 |
| Samoa | 29 | 0 | 58 | 0.000 |
| Western Samoa and Tokelau | 51 | 2 | 102 | 0.020 |
| **South America** | **7664** | **516** | **15328** | **0.034** |
| Argentina | 890 | 59 | 1780 | 0.033 |
| Bolivia | 156 | 3 | 312 | 0.010 |
| Bolivia/Chile | 20 | 1 | 40 | 0.025 |
| Brazil | 3454 | 256 | 6908 | 0.037 |
| Chile | 1056 | 49 | 2112 | 0.023 |
| Colombia | 1703 | 137 | 3406 | 0.040 |
| Ecuador | 75 | 4 | 150 | 0.027 |
| Paraguay | 17 | 0 | 34 | 0.000 |
| Peru | 293 | 6 | 586 | 0.010 |
| **Global** | **7048977** | **636852** | **14097954** | **0.045** |

Notes: Data from Allele Frequency Net Database (<http://www.allelefrequencies.net/>). USA: United States of America.

**Table S14.** *HLA-DRB1*04:01* allele frequency.

| **Continent/Country** | **Sample size** | **Pooled number of altered alleles** | **Pooled number of alleles** | **Pooled allele frequency** |
| --- | --- | --- | --- | --- |
| **Africa** | **4500** | **159** | **9000** | **0.018** |
| Algeria | 203 | 1 | 406 | 0.002 |
| Cape Verde | 124 | 5 | 248 | 0.020 |
| Central African Republic | 93 | 0 | 186 | 0.000 |
| Congo | 165 | 4 | 330 | 0.011 |
| Ethiopia | 181 | 6 | 362 | 0.016 |
| Gambia | 939 | 10 | 1878 | 0.006 |
| Guinea | 65 | 0 | 130 | 0.000 |
| Libya | 118 | 1 | 236 | 0.004 |
| Morocco | 394 | 4 | 788 | 0.005 |
| Nigeria | 258 | 1 | 516 | 0.002 |
| Rwanda | 281 | 3 | 562 | 0.005 |
| Sao Tome and Principe | 98 | 4 | 196 | 0.021 |
| South Africa | 403 | 61 | 806 | 0.075 |
| Sudan | 200 | 2 | 400 | 0.005 |
| Tanzania | 336 | 7 | 672 | 0.011 |
| Tunisia | 642 | 51 | 1284 | 0.039 |
| **Asia** | **385608** | **6950** | **771216** | **0.009** |
| Borneo | 21 | 0 | 42 | 0.000 |
| China | 14494 | 309 | 28988 | 0.011 |
| Gaza | 165 | 4 | 330 | 0.012 |
| Hong Kong | 16753 | 80 | 33506 | 0.002 |
| India | 35133 | 684 | 70266 | 0.010 |
| Indonesia | 243 | 0 | 486 | 0.000 |
| Iran | 757 | 105 | 1514 | 0.069 |
| Israel | 195657 | 3156 | 391314 | 0.008 |
| Japan | 25398 | 469 | 50796 | 0.009 |
| Jordan | 146 | 58 | 292 | 0.197 |
| Lebanon | 428 | 71 | 856 | 0.083 |
| Malaysia | 1515 | 22 | 3030 | 0.007 |
| Mongolia | 861 | 124 | 1722 | 0.072 |
| Pakistan | 389 | 7 | 778 | 0.009 |
| Philippines | 84 | 0 | 168 | 0.000 |
| Russia | 5265 | 714 | 10530 | 0.068 |
| Saudi Arabia | 29366 | 675 | 58732 | 0.011 |
| South Korea | 6640 | 84 | 13280 | 0.006 |
| Sri Lanka | 714 | 26 | 1428 | 0.018 |
| Taiwan | 49687 | 342 | 99374 | 0.003 |
| Thailand | 801 | 3 | 1602 | 0.002 |
| Turkey | 250 | 10 | 500 | 0.020 |
| United Arab Emirates | 570 | 6 | 1140 | 0.005 |
| Vietnam | 271 | 2 | 542 | 0.004 |
| **Europe** | **3641953** | **585353** | **7283906** | **0.080** |
| Armenia | 100 | 9 | 200 | 0.045 |
| Austria | 200 | 27 | 400 | 0.068 |
| Azores | 232 | 14 | 464 | 0.030 |
| Belarus | 275 | 57 | 550 | 0.104 |
| Belgium | 99 | 12 | 198 | 0.061 |
| Bulgaria | 55 | 1 | 110 | 0.009 |
| Cretan Islanders | 124 | 1 | 248 | 0.004 |
| Croatia | 4235 | 240 | 8470 | 0.028 |
| Czech Republic | 5338 | 617 | 10676 | 0.058 |
| Denmark | 55 | 19 | 110 | 0.176 |
| England | 829 | 113 | 1658 | 0.068 |
| Finland | 241 | 36 | 482 | 0.075 |
| France | 43603 | 5701 | 87206 | 0.065 |
| Georgia | 199 | 8 | 398 | 0.020 |
| Germany | 3526216 | 572617 | 7052432 | 0.081 |
| Greece | 924 | 15 | 1848 | 0.008 |
| Ireland | 1250 | 273 | 2500 | 0.109 |
| Italy | 2782 | 78 | 5564 | 0.014 |
| Kosovo | 124 | 2 | 248 | 0.008 |
| Macedonia | 410 | 11 | 820 | 0.014 |
| Madeira | 358 | 15 | 716 | 0.021 |
| Netherlands | 1816 | 269 | 3632 | 0.074 |
| Norway | 181 | 51 | 362 | 0.140 |
| Poland | 44547 | 4695 | 89094 | 0.053 |
| Portugal | 275 | 19 | 550 | 0.034 |
| Scotland | 99 | 20 | 198 | 0.103 |
| Slovenia | 240 | 0 | 480 | 0.000 |
| Spain | 6622 | 334 | 13244 | 0.025 |
| Sweden | 284 | 45 | 568 | 0.079 |
| Ukraine | 240 | 54 | 480 | 0.112 |
| **North America** | **2981119** | **275362** | **5962238** | **0.046** |
| Canada | 122 | 4 | 244 | 0.016 |
| Costa Rica | 558 | 9 | 1116 | 0.008 |
| Cuba | 78 | 2 | 156 | 0.015 |
| Jamaica | 132 | 9 | 264 | 0.034 |
| Martinique | 100 | 6 | 200 | 0.030 |
| Mexico | 1252 | 25 | 2504 | 0.010 |
| Nicaragua | 494 | 9 | 988 | 0.009 |
| Panama | 462 | 16 | 924 | 0.018 |
| USA | 2977921 | 275281 | 5955842 | 0.046 |
| **Oceania** | **1567** | **53** | **3134** | **0.017** |
| Australia | 103 | 1 | 206 | 0.005 |
| Cook Islands | 78 | 4 | 156 | 0.026 |
| Fiji | 57 | 0 | 114 | 0.000 |
| Kiribati | 62 | 0 | 124 | 0.000 |
| Nauru | 67 | 5 | 134 | 0.037 |
| New Caledonia | 65 | 4 | 130 | 0.031 |
| New Zealand | 378 | 33 | 756 | 0.044 |
| Niue | 70 | 0 | 140 | 0.000 |
| Papua New Guinea | 585 | 4 | 1170 | 0.003 |
| Samoa | 51 | 1 | 102 | 0.010 |
| Western Samoa and Tokelau | 51 | 1 | 102 | 0.010 |
| **South America** | **7361** | **289** | **14722** | **0.020** |
| Argentina | 890 | 29 | 1780 | 0.016 |
| Bolivia | 69 | 2 | 138 | 0.015 |
| Brazil | 3487 | 122 | 6974 | 0.017 |
| Chile | 941 | 22 | 1882 | 0.012 |
| Colombia | 1882 | 111 | 3764 | 0.030 |
| Ecuador | 75 | 3 | 150 | 0.020 |
| Paraguay | 17 | 0 | 34 | 0.000 |
| **Global** | **7022108** | **868166** | **14044216** | **0.062** |

Notes: Data from Allele Frequency Net Database (<http://www.allelefrequencies.net/>). USA: United States of America.

1. **Supplementary Figures**


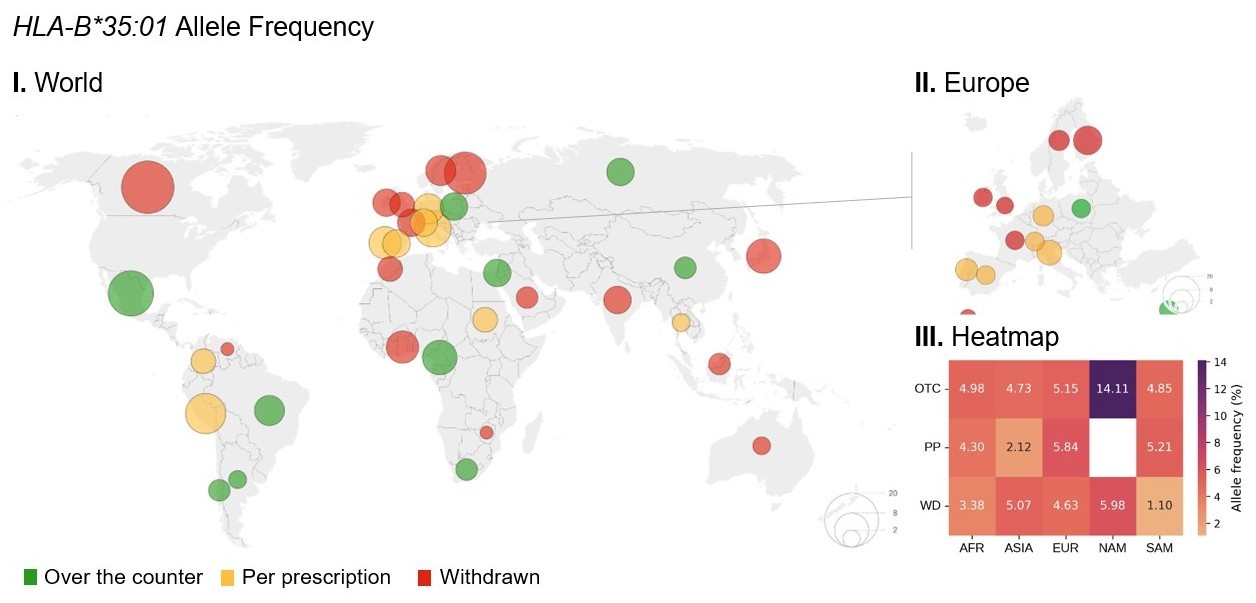


**Figure S1.** Global distribution of *HLA-B*35:01* allele frequencies in relation to metamizole legal status. **I**. World map illustrating the frequency of the *HLA-B*35:01* allele across different countries. The size of each circle is proportional to the allele frequency in that region, with the largest circles corresponding of a frequency of 20%. Circle colors indicate the legal status of metamizole in each country: green for over the counter (OTC), yellow for prescription-only (PP), and red for withdrawn (WD). **II**. Regional map of Europe showing *HLA-B*35:01* allele frequencies and metamizole legal status. **III**. Heatmap displaying the average allele frequency (%) stratified by continent—Africa (AFR), Asia (ASIA), Europe (EUR), North America (NAM), and South America (SAM)—and metamizole legal status. Data from Allele Frequency Net Database (AFND) (<https://www.allelefrequencies.net/default.asp>).


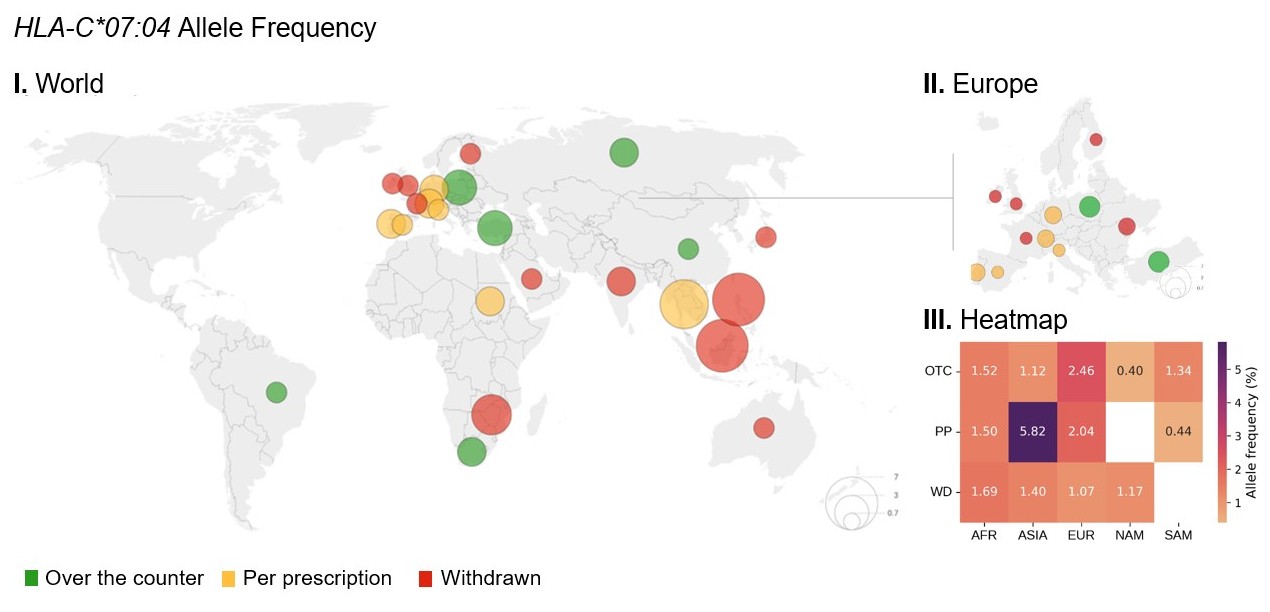


**Figure S2.** Global distribution of *HLA-C*07:04* allele frequencies in relation to metamizole legal status. **I**. World map illustrating the frequency of the *HLA-C*07:04* allele across different countries. The size of each circle is proportional to the allele frequency in that region, with the largest circles corresponding of a frequency of 20%. Circle colors indicate the legal status of metamizole in each country: green for over the counter (OTC), yellow for prescription-only (PP), and red for withdrawn (WD). **II**. Regional map of Europe showing *HLA-C*07:04* allele frequencies and metamizole legal status. **III**. Heatmap displaying the average allele frequency (%) stratified by continent—Africa (AFR), Asia (ASIA), Europe (EUR), North America (NAM), and South America (SAM)—and metamizole legal status. Data from Allele Frequency Net Database (AFND) (<https://www.allelefrequencies.net/default.asp>).


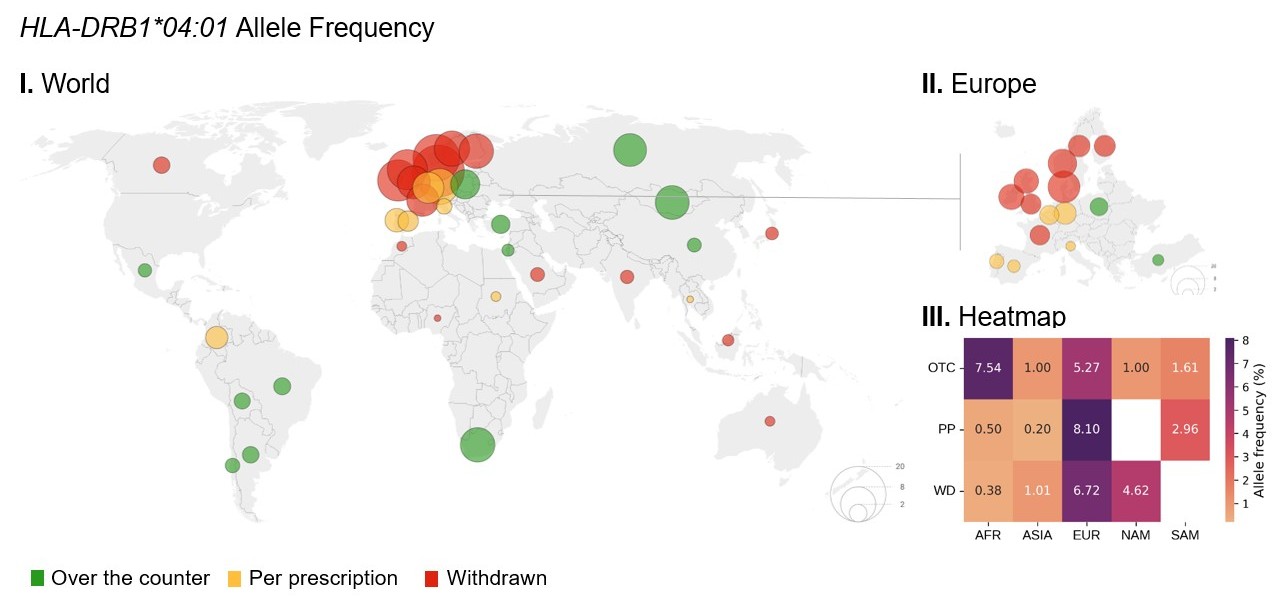


**Figure S3.** Global distribution of *HLA-DRB1*04:01* allele frequencies in relation to metamizole legal status. **I**. World map illustrating the frequency of the *HLA-DRB1*04:01* allele across different countries. The size of each circle is proportional to the allele frequency in that region, with the largest circles corresponding of a frequency of 20%. Circle colors indicate the legal status of metamizole in each country: green for over the counter (OTC), yellow for prescription-only (PP), and red for withdrawn (WD). **II**. Regional map of Europe showing *HLA-DRB1*04:01* allele frequencies and metamizole legal status. **III**. Heatmap displaying the average allele frequency (%) stratified by continent—Africa (AFR), Asia (ASIA), Europe (EUR), North America (NAM), and South America (SAM)—and metamizole legal status. Data from Allele Frequency Net Database (AFND) (<https://www.allelefrequencies.net/default.asp>).


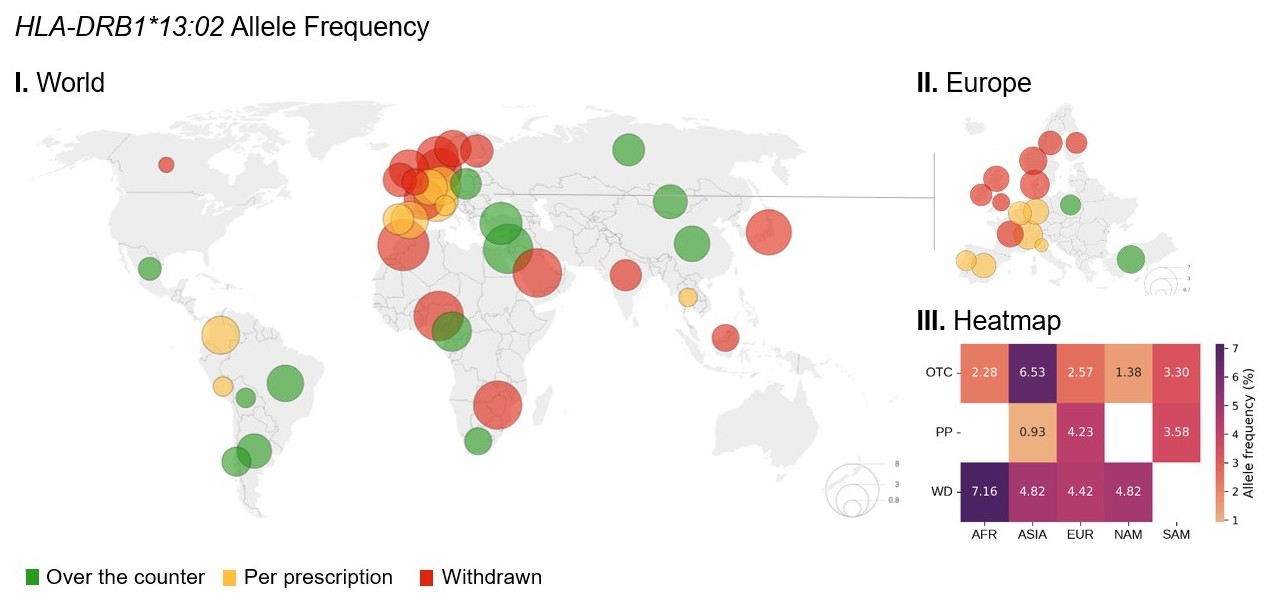


**Figure S4.** Global distribution of *HLA-DRB1*13:02* allele frequencies in relation to metamizole legal status. **I**. World map illustrating the frequency of the *HLA-DRB1*13:02* allele across different countries. The size of each circle is proportional to the allele frequency in that region, with the largest circles corresponding of a frequency of 20%. Circle colors indicate the legal status of metamizole in each country: green for over the counter (OTC), yellow for prescription-only (PP), and red for withdrawn (WD). **II**. Regional map of Europe showing *HLA-DRB1*13:02* allele frequencies and metamizole legal status. **III**. Heatmap displaying the average allele frequency (%) stratified by continent—Africa (AFR), Asia (ASIA), Europe (EUR), North America (NAM), and South America (SAM)—and metamizole legal status. Data from Allele Frequency Net Database (AFND) (<https://www.allelefrequencies.net/default.asp>).


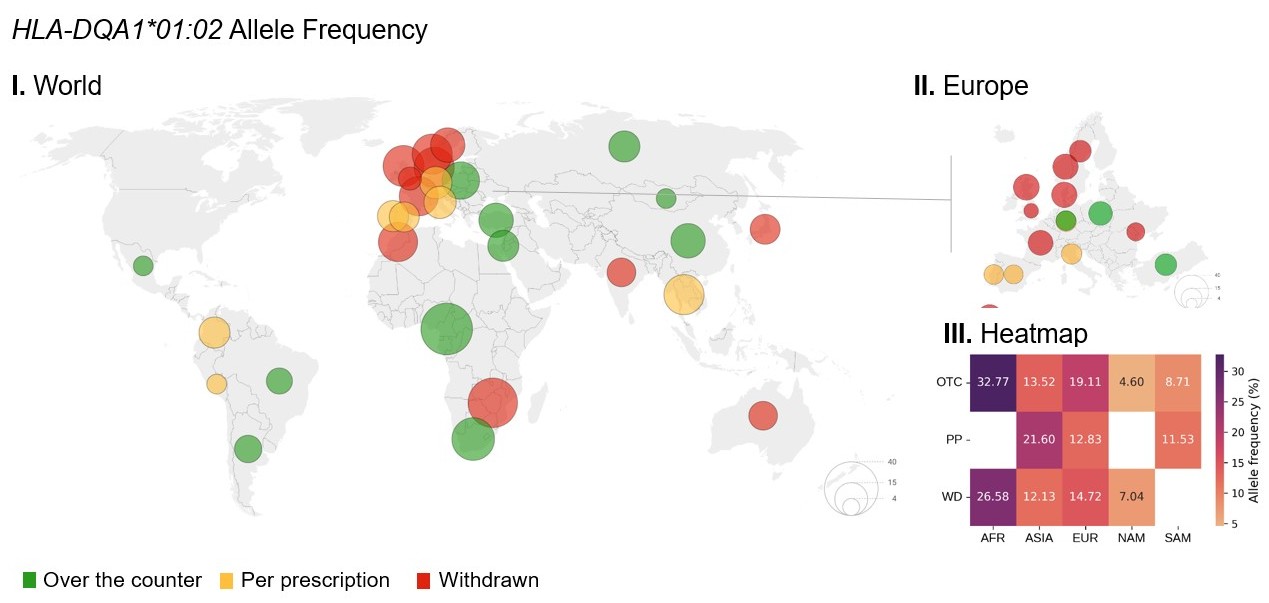


**Figure S5.** Global distribution of *HLA-DQA1*01:02* allele frequencies in relation to metamizole legal status. **I**. World map illustrating the frequency of the *HLA-DQA1*01:02* allele across different countries. The size of each circle is proportional to the allele frequency in that region, with the largest circles corresponding of a frequency of 20%. Circle colors indicate the legal status of metamizole in each country: green for over the counter (OTC), yellow for prescription-only (PP), and red for withdrawn (WD). **II**. Regional map of Europe showing *HLA-DQA1*01:02* allele frequencies and metamizole legal status. **III**. Heatmap displaying the average allele frequency (%) stratified by continent—Africa (AFR), Asia (ASIA), Europe (EUR), North America (NAM), and South America (SAM)—and metamizole legal status. Data from Allele Frequency Net Database (AFND) (<https://www.allelefrequencies.net/default.asp>).


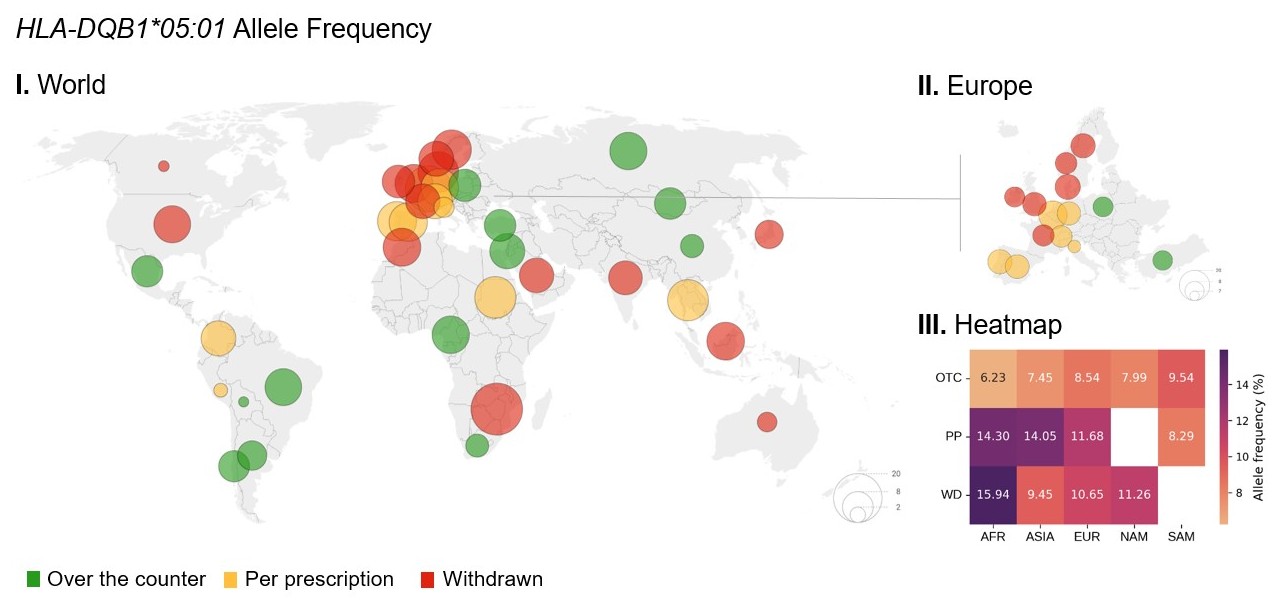


**Figure S6.** Global distribution of *HLA-DQB1*05:01* allele frequencies in relation to metamizole legal status. **I**. World map illustrating the frequency of the *HLA-DQB1*05:01* allele across different countries. The size of each circle is proportional to the allele frequency in that region, with the largest circles corresponding of a frequency of 20%. Circle colors indicate the legal status of metamizole in each country: green for over the counter (OTC), yellow for prescription-only (PP), and red for withdrawn (WD). **II**. Regional map of Europe showing *HLA-DQB1*05:01* allele frequencies and metamizole legal status. **III**. Heatmap displaying the average allele frequency (%) stratified by continent—Africa (AFR), Asia (ASIA), Europe (EUR), North America (NAM), and South America (SAM)—and metamizole legal status. Data from Allele Frequency Net Database (AFND) (<https://www.allelefrequencies.net/default.asp>).


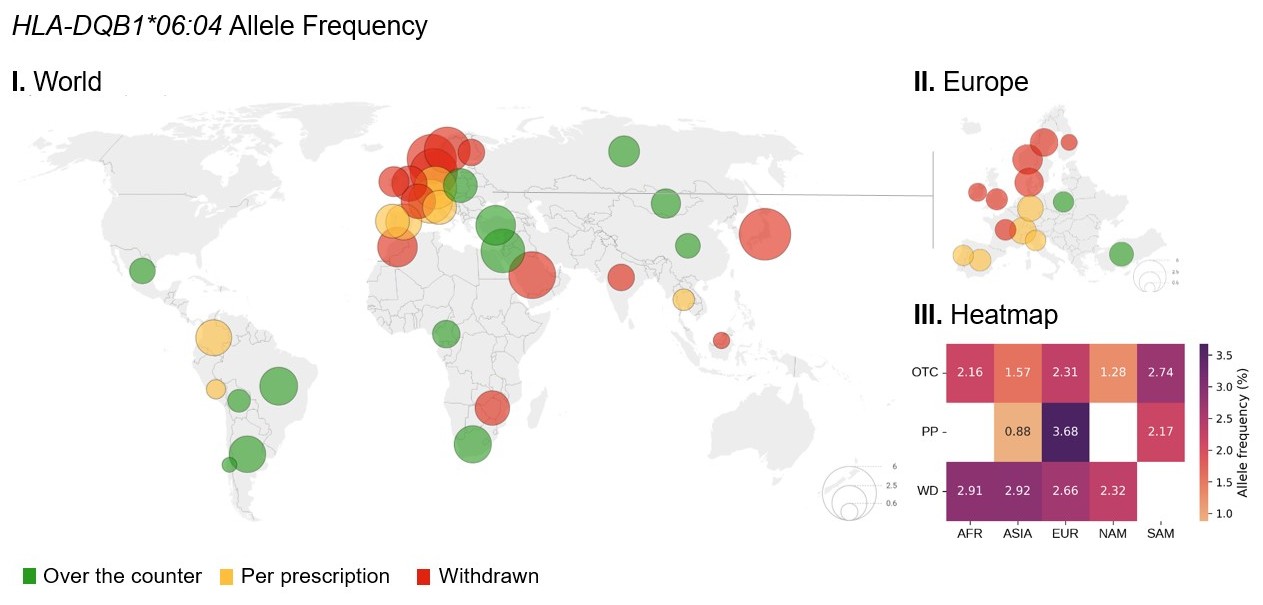


**Figure S7.** Global distribution of *HLA-DQB1*06:04* allele frequencies in relation to metamizole legal status. **I**. World map illustrating the frequency of the *HLA-DQB1*06:04* allele across different countries. The size of each circle is proportional to the allele frequency in that region, with the largest circles corresponding of a frequency of 20%. Circle colors indicate the legal status of metamizole in each country: green for over the counter (OTC), yellow for prescription-only (PP), and red for withdrawn (WD). **II**. Regional map of Europe showing *HLA-DQB1*06:04* allele frequencies and metamizole legal status. **III**. Heatmap displaying the average allele frequency (%) stratified by continent—Africa (AFR), Asia (ASIA), Europe (EUR), North America (NAM), and South America (SAM)—and metamizole legal status. Data from Allele Frequency Net Database (AFND) (<https://www.allelefrequencies.net/default.asp>).
